# The pathogenicity and virulence of Leishmania - interplay of virulence factors with host defenses

**DOI:** 10.1080/21505594.2022.2074130

**Published:** 2022-05-30

**Authors:** Anand Kumar Gupta, Sonali Das, Mohd Kamran, Sarfaraz Ahmad Ejazi, Nahid Ali

**Affiliations:** Infectious Diseases and Immunology Division, CSIR-Indian Institute of Chemical Biology, Kolkata, India

**Keywords:** *Leishmania*, macrophage, virulence factor, signaling pathways, ncRNAs, diagnosis, therapeutics, immunomodulators

## Abstract

Leishmaniasis is a group of disease caused by the intracellular protozoan parasite of the genus *Leishmania*. Infection by different species of *Leishmania* results in various host immune responses, which usually lead to parasite clearance and may also contribute to pathogenesis and, hence, increasing the complexity of the disease. Interestingly, the parasite tends to reside within the unfriendly environment of the macrophages and has evolved various survival strategies to evade or modulate host immune defense. This can be attributed to the array of virulence factors of the vicious parasite, which target important host functioning and machineries. This review encompasses a holistic overview of leishmanial virulence factors, their role in assisting parasite-mediated evasion of host defense weaponries, and modulating epigenetic landscapes of host immune regulatory genes. Furthermore, the review also discusses the diagnostic potential of various leishmanial virulence factors and the advent of immunomodulators as futuristic antileishmanial drug therapy.

## Introduction

Leishmaniasis is a multifarious vector-borne neglected tropical disease (NTD) varying in the form of disease onset and includes cutaneous (CL), muco-cutaneous (MCL), visceral (VL), and post kala-azar dermal leishmaniasis (PKDL) caused by at least 20 species of the obligate intracellular parasite, *Leishmania* [1,2]. Of these, VL is the most severe form affecting visceral organs like spleen and liver and can prove fatal if left untreated. CL and MCL forms are comparatively less severe, with the former manifesting self-healing ulcers and the latter resulting in disfiguring lesions of oro-pharyngeal mucosal linings. The disease is transmitted by female *Phlebotomine sp*. and *Lutzomyia sp*. sand flies. However, transmission of the disease is rare by syringe sharing, blood transfusions, or from mother to foetus. The World Health Organisation (WHO) enlists leishmaniasis as the second most severe NTD next to malaria and estimates over a million fresh cases annually [3]. The existence of leishmaniasis can be dated back to as early 2500–1500 B.C. based on archaeological indications including pictures, mummified bodies, and statues (Elisama et al, 2014). The detailed description of CL (termed as “Apello Boil”) was given by Alexander Russel. In 1903 Sir William Leishman and Charles Donovan provided the clinical description of VL (termed as “*Kala-azar*”) [4].

Currently, the disease is prevalent in tropical and sub-tropical countries and southern Europe and covers a geographic range of approximately 90 countries. According to WHO reports, apart from Australia and Antarctica, the disease can be found in people of every continent. In the Old World, leishmaniasis is reported in some parts of Asia, the tropical regions and northern part of Africa, southern Europe, and Middle East. In the New World, it is prevalent in some areas of Mexico, South, and Central America. The estimated number of CL per year still may range from 0.7–0 to 1.2 million. There is a decline in the number of estimated cases of the visceral form of the disease and may range from over 400,000 cases to less than 100,000 cases [3].

In the current review, we discuss about the various virulence factors of the different species of *Leishmania* and the strategies exploited by the parasites to overcome the immune defense mechanisms of the host for successful infection establishment. The review also highlights the current scenario of diagnosis, limitations of frontline drugs, and the therapeutic potential of immunomodulators in controlling the menace of leishmaniasis.

## Life cycle, vector, and epidemiology

*Leishmania* is a group of protozoan parasites belonging to the Class Kinetoplastae and Order Trypanosomatida, which avails a digenetic life cycle involving an insect vector and a mammalian host. *Leishmania sp* can be characterized by the two prevailing forms, the elongated (10–20 μm) motile promastigote form and the oval-shaped (3–7 μm in diameter) non-motile amastigote form. The promastigote form exists in the sand fly vector, where it undergoes various differentiation steps and transforms into the infective metacyclic promastigote form. These metacyclics are transmitted to mammalian hosts during the bite of the sand fly [5]. Amastigotes are the forms within the mammalian host, especially in phagocytic cells where they survive as intracellular parasites. When a sand fly vector bites an infected mammal, it ingests the amastigotes, which transform into the flagellated promastigote form on reaching the midgut of the insect. Eventually, the promastigotes move to the alimentary tract of the insect where they survive extracellularly and multiply by binary fission. The promastigotes then migrate towards the salivary glands and oesophagus and are later transmitted along with the insect saliva to the mammalian host during the next blood meal. The anticoagulant present in the saliva of the sand fly helps in the transmission by preventing the blood from coagulating at the site of insect bite. Post entry into the host, the promastigotes are readily taken up by macrophages or dendritic cells within which they revert back to the amastigote form and proliferate. This is followed by the eventual egress of the amastigotes from the host cells due to the host immune response-mediated cytolytic environment [6]. The amastigotes released are either phagocytosed by other macrophages or taken up by the sand fly during a blood meal. Thus, the parasite continues its life cycle and culminates in infection of surrounding cells and tissues in CL and organs rich in macrophages like the bone marrow, liver, and spleen in VL [7].

## Visceral leishmaniasis

VL also called Kala-azar or black fever is the most severe form of leishmaniasis where visceral organs like bone marrow, spleen, and liver are affected and can be fatal if not treated. The causative agents of VL in the Old World include *L. donovani* and *L. infantum,* while the major causative agent in the New World is *L. chagasi*. Clinical symptoms of the disease include fever, weight loss, anemia, hyper-gammaglobulinemia, and hepatosplenomegaly mainly due to increased parasite burden in these visceral organs. Hypersecretion of adrenocorticotropic hormone in VL patients leads to skin blackening of the patients, giving the disease its local name in India as Kala-azar [8]. Although majority of the cases are asymptomatic during the initial phase of infection, symptoms may develop even years later when the patients become immunocompromised [9]. The emergence of HIV/VL co-infection has lately been a cause of great concern [10].

## Post kala-azar dermal leishmaniasis

Treated VL patients often develop a complication of the skin, which acts as a reservoir of the parasite called PKDL. PKDL is characterized by macules, macupapules, and nodular rashes in recovered VL patients. The rashes usually develop around the mouth and gradually spread to other parts of the body. It is prevalent in areas where *L. donovani* is the causative VL agent, like in Sudan and India with 50% and 5–10% cases, respectively. Although PKDL is usually not seen in people infected with *L. infantum*, it is often reported in immune-compromised patients [11]. The manifestations of PKDL in India have been reported to occur 2–3 years post VL, while this interval is just 0–6 months in Sudan [12–14]. Recent reports associate PKDL with Th1 immune response, especially the production of interferon γ, along with IL-10 in the peripheral blood of treated VL patients.

## Cutaneous and mucocutaneous leishmaniasis

CL is the most common form of leishmaniasis, and unlike VL, it is less severe and usually self-healing. The clinical manifestations of the disease include chronic ulcers at the sites of insect bite, which often leave life-long scars, leading to social stigmas, cosmetic morbidities, and psychological effects. The extent of symptoms varies depending on the leishmanial species causing the infection and the immune state of the host. *L. major, L. aethipica,* or *L. tropica* are the species of *Leishmania* responsible for CL cases in the Mediterranean region, America, central Asia, and Middle East. In South America, the common causative agents of CL include *L. braziliensis, L. guyanensis, L. panamensis, L. mexicana,* and *L*.amazonensis [15]. Apart from human CL, canine CL is common in South America, and *L. braziliensis* and *L. chagasi* are the usual causative agents [16,17]. According to WHO, over 95% new cases of CL in 2017 were reported from just six countries, Brasil, Iran, Colombia, Afghanistan, Syria, Iraq, and Algeria.

MCL is a rare and severe variant of CL caused by *L. braziliensis, L. amazonensis,* and *L. mexicana*. It is characterized by mucosal lesions, which lead to partial or complete destruction of mucosal linings of the nose, throat, and mouth. These symptoms can be attributed to the hyperallergic immune response targeting host tissues. Like VL, MCL is life-threatening with massive lesions, which lead to permanent disfigurement requiring early diagnosis and rapid treatment. Cases of MCL are endemic in regions of Latin America, specially Bolivia, Brasil, Ethiopia, and Peru.

## Virulence factors of parasite

**Figure 1.**
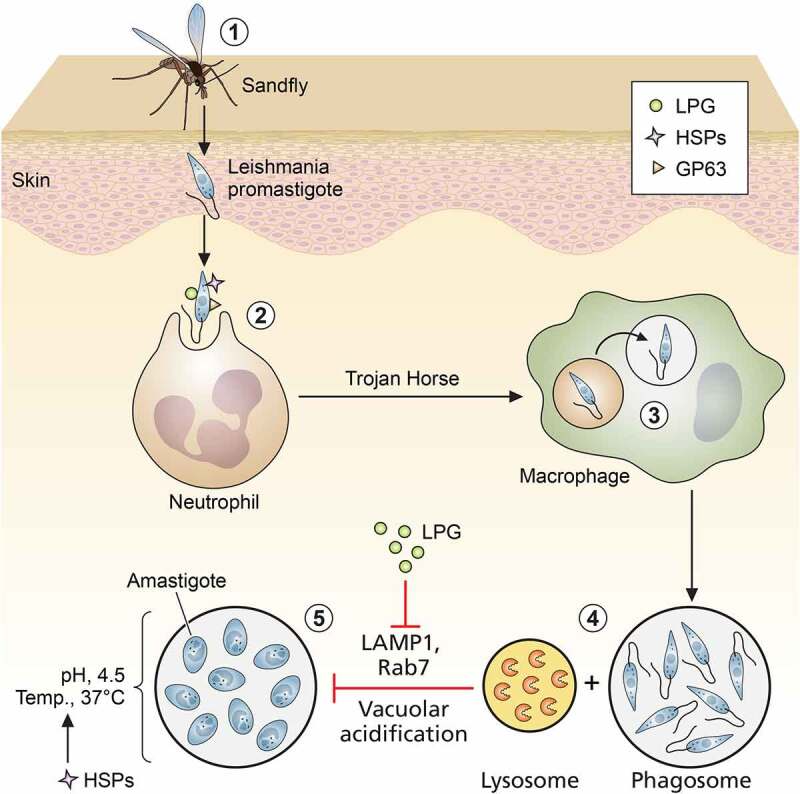
**Role of leishmanial virulence factors in entry and trans-differentiation of parasites**. (1) Entry of promastigotes through skin via bite of sand fly, (2) uptake of promastigotes by neutrophils, (3) safe transport of promastigotes from neutrophils to macrophages by “Trojan Horse” mechanism, (4) formation of parasitophorus vacuoles (PVs) by prevention of phago-lysosomal fusion, (5) trans-differentiation of promastigotes to amastigotes inside PVs (LPG: lipophosphoglycan; HSPs: heat shock proteins; and GPI: glycosyl phosphatidyl inositol). Image created through paid version of Biorender.

Evasion of macrophage immune sentinel is a well-established strategy in obligate parasites. *Leishmania sp*. has evolved with a plethora of membrane-bound or secreted virulence factors, which help in breaching the host immune barrier (Figure 1 and Table 1). Below is the summarized overview of some well-illustrated leishmanial virulence factors, which are portrayed either as potential therapeutic targets or as vaccine candidates.

**Table 1.** List of genetically modified *Leishmania* sp. virulence factors.

Name of virulence factors	Genetically modified type (heterozygous/null mutation)	Method of genetic modification	Parasite species
Lipophosphoglycan (LPG)	*lpg*-/-	Targeted gene disruption	*Leishmania major* [25,31]
GRP94	*lpg3*-/-	Targeted gene disruption	*Leishmania donovani* [45]
Arginase	*arg*-/-	Targeted gene disruption	*Leishmania donovani* [48]
EF1α	-	-	-
GP63	*gp63*-/-	Selective knock down by anti-sense RNA, Targeted gene disruption	*Leishmania amazoniensis* [74], *Leishmania major* [18]
CPC-A/B/C	*Δcpa, Δcpb, Δcpc*	Targeted gene disruption	*Leishmania mexicana* [102]
Oligopeptidase B (OPB)	*opb*-/-	Targeted gene disruption	*Leishmania donovani* [109],*Leishmania major* [110]
Deubiquitinase	*DUB2*-/-	DiCre inducible gene deletion system	*Leishmania mexicana* [114]
HSP100	*HSP100*-/-	Targeted gene disruption	*Leishmania donovani* [117],*Leishmania major* [118]
HSP78	*HSP78±* and conditional *HSP78-/-*	Targeted gene disruption, CRISPR-Cas9	*Leishmania donovani, Leishmania mexicana* [120]
sHSPs	*HSP23*-/-	Targeted gene disruption	*Leishmania donovani* [122]
A2	*A2*-deficient	Selective knock down by anti-sense RNA	*Leishmania donovani* [129]
PTP1	*PTP1*-/-	Targeted gene disruption	*Leishmania infantum* [133]

## Lipophosphoglycans

Procyclic and metacyclic promastigotes differ in the thickness of their surface glycocalyx, which is mostly composed of glycosylated proteins and glycans. Lipophosphoglycans (LPGs) are the dominant structure component of *Leishmania* surface glycocalyx [19]. LPG is synthesized in promastigotes, and its ultrastructure is composed of repetitive disaccharide units and phosphate, which are linked with a glycosyl phosphatidylinositol (GPI) anchor. Side chain composition and core positioning of LPG in surface glycocalyx largely varies between promastigotes and amastigotes. Compared to procyclics, LPG in metacyclics is longer in length and branched and completely absent in amastigotes [20,21]. Therefore, the main requirement of LPG is confined solely during parasite entry and initial infection stages. On entry, the first phagocytic cell that *Leishmania* encounters is the poly morpho-nuclear cells (PMNs) or neutrophils, where it resides as promastigote. These PMNs help in transferring the promastigotes safely inside macrophages by forming apoptotic bodies. This method is well-known as “Trojan Horse” mode of transfer, where LPG plays a crucial role [22]. LPG assists in the entry of *Leishmania sp*. promastigotes inside macrophages via binding with complement receptor CR3 and integrin receptor p150/95 [23]. After phagocytosis in a macrophage, LPG^–^/^–^ promastigotes are rapidly cleared compared to WT *L. donovani*. Moreover, external administration of purified LPG prevented the early clearance of LPG-deficient promastigotes. Thus, LPG is a highly essential surface antigen, playing a protective role during early trans-differentiation of promastigotes to amastigotes. LPG^–^/^–^ *L. major* parasites displayed compromised virulence in human dendritic cells, murine macrophage cell lines, and murine models and a poor survival rate in sand fly gut. Virulence and survival of LPG^–^/^–^ parasites was restored upon cosmid-based complementation of LPG encoding exons [24,25]. In *L. major* metacyclics, LPG is highly branched and complex compared to procyclics. Resistance against complement receptor is positively correlated with the branching of LPG [26]. *L. major* LPG prevents accumulation and lysis by C5b-C9 subunits [27]. In addition to complement and integrins, LPG helps in mannose receptor-mediated entry of parasites via its own mannan residues [28]. LPG helps in silent phagocytosis of parasites via C-reactive proteins (CRPs), without triggering the usual CRP-mediated inflammatory signaling of macrophages [29].

Upon entry, the parasite prevents fusion of phagosome and lysosome to maintain a comparatively benign microenvironment of phagosome where it can trans-differentiate from pH-sensitive promastigotes to pH-resistant amastigotes [30]. The major role of LPG as a leishmanial virulence factor was first established when Spath *et al.* generated *lpg^–^/^–^ L. major* parasites, which showed attenuated infectivity in *in vitro* and *in vivo* infection models [31]. LPG mainly prevents this fusion by chelating calcium ions (Ca^2+^) and inhibiting diacyl glycerol (DAG)/protein kinase C (PKC) signaling [32]. Purified LPG can reportedly block PKCα signaling of BALB/c and C57BL/6 mice macrophages [33]. Another major breakthrough in *Leishmania* infection biology came with the discovery of the potential role of LPG in delaying the appearance of late endosomal markers, Rab7 and LAMP-1, on parasitophorus vacuoles (PV) and F-actin accumulation [34]. LPG not only prevents vacuolar acidification but also safeguards the parasites from reactive oxygen intermediates (ROIs) and lysosomal enzyme-mediated damages [35]. The strong negative charge and the presence of disaccharide repeats like galactose–mannose in LPG mainly help in inhibiting lysosomal enzymes [36]. Additionally, LPG can block the NADPH oxidase assembly in phagosomes, which significantly affects the activation of innate immunity during parasite infection establishment [37]. Although LPG is absent in amastigotes, LPG predesigns an immune-suppressed situation during parasite entry so that the amastigotes can easily survive and proliferate within. Interaction of parasite surface LPG with macrophage suppresses IL1β and IL12a secretion, as well as IFN-γ-mediated nitric oxide (NO) generation [25,38]. Besides Ca^2+^, LPG alters other secondary messengers of host macrophage like inositol lipids and inositol phosphate, which prevent phagocytosis and NO production [32,39]. In addition to the secondary messengers, LPG also prevents phosphorylation of ERK1/2 and subsequent activation of NF-κB and AP-1 module in order to inhibit NO generation [40]. LPG may not be essential for all *Leishmania sp*. for their virulence [41], but in some species of *Leishmania*, LPG seems to play a crucial role for virulence and intracellular survival (Figure 1).

LPG was identified as a potential vaccine candidate for VL and is the first leishmanial antigen, which was conjugated with poly-electrolytic delivery vehicle named polyacrylic acid (PAA). LPG-PAA complex showed minimal toxicity in J774 and mouse peritoneal macrophage cells and exhibited enhanced anti-leishmanial efficacy [42,43]. Purified *L. mexicana* LPG administration in mice confirmed that LPG can induce PD-1 expression on the surface of CD8+ T cells and PD-L2 expression upon the macrophage surface, which induce immune-suppressive signals during vaccination [44]. However, LPG achieved success rate in vaccination due to low immunogenicity.

## GRP94

Genetic complementation of LPG-defective *L. donovani* parasites led to the identification of a unique and truncated form of LPG, which contains only Manα1-PO_4_ residue of first repeat unit of LPG disaccharide. This subtype of LPG was termed as LPG3, and it shared structural homology with mammalian endoplasmic reticulum (ER) chaperone GRP94 [45]. Like mammalian homolog, parasite LPG3/GRP94 is also localized in the parasite ER and regulates chaperone-like functions, i.e. protein assembly, secretion, antigen presentation, etc. [46]. Interestingly, null mutation of GRP94-induced pleotropic defects such as downregulation of surface GPI-anchored proteins with no effect on either N-glycosylated protein synthesis or proliferation rate of the promastigotes. Expression of GRP94 is not dependent on stress and is mainly regulated developmentally. Orthologs of *L. infantum* GRP94 were found to be highly immunogenic, indicating the role of GRP94 in regulating the immune responses of host during infection. Altogether, LPG3 plays a completely different and exclusive role in leishmania metabolism compared to its mammalian homolog [45].

## Arginase

Besides being part of the Krebs-Henseleit cycle, L-arginine aminohydrolase or Arginase plays non-canonical role in infection persistence and virulence of *Leishmania sp*. Arginase is a metalloenzyme, which hydrolyses L-arginine into L-ornithine and urea, which contributes to the ureotelic behavior of *Leishmania*. In order to survive inside the macrophage and transform into amastigote form, it is crucial for *Leishmania sp*. to overcome the toxic effect of host nitric oxide. The parasite, apart from exploiting the host arginase, itself possesses its own arginase. Parasite arginase converts host L-arginine into L-ornithine [47] and helps in bypassing the L-arginine pool from nitric oxide generation toward L-ornithine production, which is used for the survival of the amastigotes. Parasite arginase is mainly localized in the glycosomes of both the promastigotes and amastigotes and gets trafficked inside the host whenever there is a lack of host arginase pool [47]. Arginase not only helps in maintaining the L-arginine pool inside promastigotes but also helps in the survival of amastigotes inside macrophages, contributing to parasite virulence. The potential of parasite arginase as a virulence factor has been further confirmed by infectivity assay with *arg*-/- *L. major* parasites. Knockout parasites showed impaired survival potential both *in vitro* and *in vivo* and resulted in decreased intracellular parasite numbers [48].

## EF1α

The quest for other leishmanial virulence factors mediating regulation of macrophage signaling led to the advent of another essential virulence factor, elongation factor 1α. Eukaryotic EF1α is a GTP-dependent translation factor, which mainly catalyzes binding of amino-acyl tRNAs with ribosome. Structural modeling demonstrated that compared to human EF1α, 12-amino acid long-loop region is absent from leishmanial EF1α. This opened a new avenue for structure-based drug targeting, like targeting of this particular domain of parasite EF1α [49]. EF1α mainly gets exported from PVs and binds to host SHP-1 phosphatase, thereby leading to macrophage inactivation. EF1α lacks N-terminal secretory peptide, suggesting that EF1α is secreted out of the PVs via a non-classical ESCRT-III pathway [50]. Interestingly, only leishmanial EF1α, and not human EF1α, can block macrophage SHP-1 activity, which offers further areas of structural investigations. Compared to GP63, EF1α offers higher potency to block the SHP-1, preventing NO generation in response to IFN-γ stimulation [51]. EF1α is a part of the leishmanial secretome and functions as a cargo for exosomal export of other leishmanial antigens from PVs to macrophage cytosol [52,53]. Besides being a potential drug target, EF1α is a potential vaccine candidate. Sabur *et al.* reported that cationic liposomal EF1α can trigger delayed-type hypersensitive (DTH) response, T_helper_ cell proliferation, augmenting IFN-γ response, and long-term protective memory response of both CD4+ and CD8+ T cells in *L. donovani*-challenged BALB/c mice [54].

## Proteases

Proteases are a class of enzymes that can digest the target proteins or peptides, manifesting important roles in the life cycle of any organism. These proteases are classified based on the amino acids present in their active site, like serine-, threonine-, aspartyl-, metallo-, and cysteine proteases. In *Leishmania sp.,* aspartyl-, serine-, metallo-, and cysteine proteases have been extensively studied as virulence factors [55–57]. Expression of active aspartyl protease in the soluble fraction of *L. mexicana* promastigotes was found to be essential for parasite proliferation, but its role in host modulation is still not well deciphered [58].

### GP63

GP63, discovered in 1980, is a 60–66 kDa molecular weight leishmanial protease and was considered as a major surface antigen (MSA) [56]. Later, due to its ability of binding with Concanavalin A (Con A) and high glycosylation, it was renamed GP63. GP63 is also known as leishmanolysin. Besides *Leishmania*, GP63 shares structural homologs in *Trypanosoma sp*. and *Trichomonasvaginalis* [59]. GP63 has a wide range of substrate specificity, including gelatin, hemoglobin, albumin, fibrinogen, and casein. It mainly cleaves at the junction of hydrophilic and hydrophobic amino acid residues at positions P1 and P’1, with basic amino acid residues at positions P’2 and P’3 [60]. GP63, a Zn-dependent metalloprotease, belongs to the metzincin class. Presence of the sequence motif HExxHxxGxxH and a pro-peptide at its N-terminal end makes it a zymosan/pro-enzyme-like molecule that remains inactive after its translation [59]. During trans-differentiation from promastigotes to amastigotes, expression of GP63 drops [61]. However, in amastigotes, the low expression of GP63 is compensated by the low expression of LPG; hence, GP63 is no longer buried [20]. Like other secretory proteins, GP63 is also processed in the endoplasmic reticulum, and nearly 75% of GP63 is either expressed on the parasite surface or is a part of the lipid rafts [62,63]. Extent of glycosylation, anchoring with glycosyl phosphatidylinositol (GPI) link, Zn-chelation, and auto-proteolysis are the main factors that regulate secretion of GP63 [64,65]. gp63^–^/^–^*L. amazonensis* confirmed that GP63 can be secreted either via vesicles or directly. Direct secretion from a cell surface is dependent upon autoproteolytic cleavage of the inactivation peptide. In *L. chagasi* and *L. donovani,* large micelle-based and exosome-based secretion of GP63 takes place. Thus, despite N-terminal secretory signal peptide, GP63 can get secreted via the ESCRT-III-dependent non-conventional pathways [66,67] (Figure 1).

GP63 contributes to *Leishmania* virulence by proteolytically cleaving C3b into C3bi. C3b is essential for the recruitment of complement lysis machinery via the CR1 receptor signaling of macrophages. C3bi thus not only hampers the complement-mediated lysis of parasites but also augments safe internalization of parasites via C3bi opsonization into macrophages [68,69]. In addition to CRs, GP63 also assists in the parasite adherence to macrophage through fibronectin receptor (FR) [70]. GP63 can degrade fibronectin, thus helping in downregulating ROS generation and supporting parasite survival in macrophages [71]. GP63 is one of the major virulence factors that helps in the degradation of extracellular matrix of subcutaneous tissue and helps in tissue penetration and dissemination of *L. mexicana* [72].

The role of GP63 in suppressing macrophage immune signaling is controversial, but it aids in infection persistence. Interestingly, GP63-coated PVs are resistant toward phago-lysosomal degradation [73]. Antisense RNA-silenced GP63 *L. amazonensis* exhibited lower parasite burden, confirming a major role of GP63 in intracellular amastigote survival [74]. Additionally, GP63 can cleave the SNARE-Vamp8 protein in order to prevent phagosomal maturation and antigen cross-presentation to CD8+ T cells [75]. GP63 is directly involved in leishmanial hijacking of diverse host macrophage immune signaling machineries. MARCKS (myristoylated alanine-rich C kinase substrate) are the major inflammatory mediators that normally get up-regulated in LPS-stimulated macrophages. MARCKS results in the activation of MARCKS-regulated proteins (MRP), which function as the major substrate of PKCs. These MRPs are proteolytically cleaved by GP63 [76,77]. *L. major*-infected macrophages resulted in exhausted levels of MRP, which was restored on treatment with GP63 inhibitors [76]. Amastigotes generally lack LPG and safeguard themselves from ROI-mediated damage via GP63-mediated prevention of PKC activation [77]. Protein tyrosine phosphatases (PTPs) like SHP-1 get activated immediately upon *Leishmania* entry, which negatively regulate the activation of inflammatory JAK2/STAT1α pathway [78]. GP63 after entering the macrophages through lipid rafts can transactivate PTPs (SHP-1, TCPTP, and PTP1B) by cleaving their C-terminal portion [79]. Besides JAK/STAT signaling, *L. donovani* negatively regulates TLR4-signaling-mediated NO generation by SHP-1. SHP-1 inactivates IRAK-1 by tyrosine dephosphorylation and blocks TLR4 pathway-mediated activation of NO by IRAK1/IRAK4 module [80].

In addition to STAT1α, *Leishmania* GP63 exerts control over other major transcription factors of macrophages like NF-κB and AP-1. It enters the nuclear matrix via lipid rafting and ceramide augmentation of the nuclear membrane. GP63 specifically and partially degrades NF-κB subunit p65RelA and produces p35RelA, which heterodimerizes with p50RelB and induces disease-promoting chemokines like macrophage inflammatory protein (MIP) 1α and MIP1β [81,82]. Like JAK2/STAT1, AP-1 (composed of C-jun and C-fos) is also essential for IFN-γ-mediated NO production by macrophages [40]. *Leishmania sp*. induces GP63-mediated proteolytic degradation of C-Jun subunit of AP-1. In addition, it down-regulates IFN-γ signaling, as well as subsides IFN-α/β transcription by targeting mTOR. GP63 cleaves mTOR, leading to dephosphorylation of 4E-BP1, which hampers the CAP-dependent translation of IFN-α/β in macrophages infected with *L. major* promastigotes [83].

Inflammasome activation plays a crucial role in destroying intracellular amastigote burden. Inflammasome complex is mainly initiated by NOD-like receptor protein 3 (NLRP3), which gets two subsequent signals – i) TLR agonists like LPS and ATP and ii) intracellular ROS [84]. Activation of NLR3 receptor complex leads to the production of IL-1β and IL-6, which assist in the clearance of parasites. Exosomal GP63 of *L. major* parasites reportedly blocks ROS production by preventing PKC activation and degrades NLP3 inflammasome complex to prevent activation of IL-1β in both murine and human infection models [85].

Interestingly, GP63 becomes a standalone vaccine candidate because it is essential for parasite survival, highly immune reactive surface antigen, and low mutagenic [86]. Amino acid sequences of human T cell epitope have been identified in *L. major, L. donovani,* and *L. chagasi* GP63 exon sequences [87–89]. These epitopes can potentially mount *Leishmania*-specific CD8+ T cell response and elevate IFN-γ levels, which can offer excellent protection [87]. Whole exon of *L. major* GP63 became the first candidate for DNA vaccine [86]. Later on, polytope DNA vaccine with multiple T cell epitopes of GP63 and HSP70 from *Mycobacterium tuberculosis* adjuvant proved successful in *L. donovani*-infected BALB/c mice model [90]. GP63 was also employed as a candidate of choice for novel gunshot emulsification-based immunization approach and provided better protection against *L. mexicana* in infected BALB/c mice compared to Soluble Leishmania Antigen (SLA) [91]. Additionally, recombinant Ldgp63 containing cationic liposome acted as a stable and potent antigen, which induced long-term memory responses in BALB/c mice against *L. donovani* infection [92].

### Cysteine proteases

Cysteine proteases (CPs) of *Leishmania sp*. are being studied as effective drug target and vaccine candidates. CPs that are found in *Leishmania* sp. have similar mode of action as papain proteases and are subdivided into three types, CPA, CPB, and CPC. Among these, CPA and CPB are cathepsin L-like enzymes and CPC is a cathepsin B-like enzyme [93–95]. Interestingly, in *L. donovani* and *L. major*, a single nucleotide polymorphism (SNP) has been observed in the genes encoding CPs, which determines whether the parasite infection will be dermatropic or viscerotropic [95,96]. The role of CPs in *Leishmania* virulence was perceived from murine models infected with parasites belonging to the *L. mexicana* complex such as *L. mexicana, L. pifanoi*, and *L. amazonensis* [97–99]. Besides this, a positive correlation was observed between the expression level of CPs and parasite virulence in hamsters infected with *L. infantum* and human cell lines infected with *L. chagasi* [57,100]. Expression of CPs was significantly enriched in *L. amazonensis* amastigote extract compared to promastigotes, which further strengthened the fact that the CPs play a potential role in the survival of amastigotes inside hosts [57]. Additionally, *L. tropica* parasites, treated with CP inhibitor, exhibited diminished growth rate, pathogenicity, and survival [101]. Infectivity analysis with *Δcpa, Δcpb,* and *Δcpc L. mexicana* parasites demonstrated that parasite virulence was severely hampered after deletion of *cpb* gene compared to *cpa* and *cpc* gene. Interestingly, parasite virulence was completely restored by not single but complementation with multiple copies of *cpb* in cosmid vector [102]. *Δcpb L. mexicana* parasites were reportedly found to be unable to induce IL-4 expression in BALB/c mice, and a Th1 response was mounted limiting their expansion. However, insertion of *cpb* gene recovered the virulence and the capacity of the parasite to induce IL-4 production, indicating that *L. mexicana* infectivity in BALB/c mice is directly related with its capacity of IL-4 induction. On the contrary, in C57BL/6 mice, *Δcpb L. mexicana* parasites were unable to induce the suppression of IL12p40 and STAT4, resulting in Th1 response-mediated parasite clearance [102]. CPB assists in infection establishment of *L. major* parasites in C57BL/6 mice via suppression of IFN-γ expression and in macrophages and dendritic cells via suppression of IL-12 production [103]. Being a potential protease, CPB mainly cleaves inflammatory transcription factors like NF-κB p65 subunit, STAT-1 and AP-1, which helps the parasite in the prevention of IL-12 and NO production by the host. Unlike gp63, CPB completely cleaves p65. Besides transcription factors, CPB cleaves MHC-II protein inside the PVs, which helps in preventing antigen presentation and activation of the Th1 immune response [104]. Subcutaneous introduction of recombinant leishmanial CPB in BALB/c mice footpads augmented IL-4 and IL-5 levels with concomitant cleavage of CD25 receptor [105]. Structural insights of *Lmx*CPB confirmed that it has a COOH-terminal extension (CTE), which gets hydrolyzed before its secretion. This CTE domain also possesses immune regulatory function in the host. Later, it was established that only the CTE portion of CPB protein is capable of inducing Th2 cytokines. *L. pifanoi* and *L. amazonensis* amastigotes, pre-incubated with anti-CTE antibodies, showed decreased proliferation inside macrophages and immune suppression capabilities [97,106,107]. These data indicate that not only the active site but also the CTE domain of CPB is crucial for *Leishmania* infection establishment and host immune modulation. Besides CPB, CPC has been well studied as a potential DNA vaccine candidate against VL. CPC expressing DNA vector (pVAX1-*cpc*) induced strong immune protective Th1-biassed response in *L. donovani*-challenged BALB/c mice in association with substantial reduction of parasite burden [108]. Taken together, besides being a vital virulence factor, CPs can be potential vaccine candidates for leishmaniasis.

### Serine protease

Oligopeptidase B (OPB), a serine protease (SP) of 115 kDa molecular weight, was initially considered to have a promising role in virulence. OPB gets upregulated during amastigote stage differentiation and helps in the protection of amastigotes by covering with enolase and plasminogen for their proliferation. Role of OPB in host macrophage global gene dysregulation was established by infecting them with OPB-deficient *L. donovani* parasites [109]. Compared to WT strain that induced changes in 23 macrophage genes, OPB–/– *L. donovani* induced 495 genes. At the same time, OPB-deficient *L. major* promastigotes showed impaired development of metacyclic promastigotes and, thus, the inability to infect macrophages [110]. Activity and expression of another leishmanial SP, subtilisin protease (SUB), also increased in the amastigotes stage of *L. donovani* [111]. Mice injected with *L. amazonensis* soluble fraction containing active SP showed enhanced sensitivity toward parasite infection. On the contrary, when animals were injected with SP inhibitor-treated extract, the susceptibility of the animals toward infection diminished, suggesting a direct role of parasite SP invirulence [112].

As *Leishmania* parasites persist in both sand fly vector and mammalian hosts, they are well-equipped with some unique group of chaperons and post-translational modifiers, which assist in the transformation of the parasite from sand fly stage to mammalian stage and vice versa. Therefore, in the following section, we have assembled few such crucial proteins of parasites, which are recently getting highlighted as potential virulence factors and chemotherapeutic targets or vaccine candidates.

## Deubiquitinases (DUBs)

During trans-differentiation of parasites, reversible post-translational modifications (PTMs) like ubiquitination/deubiquitination play significant roles. Ubiquitination is a highly conserved process throughout evolution and mainly helps in maintaining the protein balance in the eukaryotic cells. In *L. mexicana* promastigotes, ubiquitination takes place with the help of 2 E1 ubiquitin-activating enzymes, 13 E2 ubiquitin-conjugating enzymes, and 79 E3 ubiquitin ligase-mediated ubiquitin-proteasome system (UPS)-based tagging and degradation of target parasite proteins. UPS pathway plays crucial roles in parasite autophagy, DNA repair, and protein trafficking. However, the key player of UPS system is deubiquitinases (DUBs), which remove the reversal ubiquitin group as and when required and add another level of fine-tuning in the parasite life cycle regulation. Till date, 20 DUBs were identified in *L. mexicana,* which mainly belong to seven structural super families, ubiquitin-specific proteases (USPs, family C19), C-terminal hydrolyases (UCHs, family C12), ovarian tumor proteases (OTUs, family C65), JAB1/MPN/MOV34 metalloenzymes (JAMM/MPN+, family M67), Josephins (family C86), MINDY (family C115), and ZUFSP (zinc finger with UFM1-specific peptidase domain protein, family C78) [113]. By employing bar-seq CRISPR-Cas9 technology, Damianou *et al*. identified DUBs 4, 7, and 13 as essential during amastigote stage development of *L. mexicana* promastigotes. Additionally, by chemical probing, they deciphered essentiality of DUB 3, 5, 6, 8, 10, 11, and 14 for parasite survival during *in vivo* infection in mice. With the help of DiCre inducible gene deletion system, Damianou *et al*. also demonstrated the importance of DUB2 in the establishment of infection in macrophages and animals [114]. DUB2 mainly cleaves off di-ubiquitine chain in a broad linkage-specific manner. The association of another DUB, Otubain (OTU), with host immune signaling was first established during *L. infantum* infection by Azevedo *et al*. OTU mainly cleaves K48-linked tetra-ubiquitin chains of target proteins [115]. Recombinant LiOTU can trigger inflammatory responses in host macrophages via lipid droplet biogenesis, IL-6, and TNFα. Interestingly, a recent report highlighted a highly sensitive diagnostic potential of *L. donovani* OTU for endemic VL samples [116]. These reports collectively suggest the fervent role of DUBs in the virulence of parasite.

## Heat shock protein (HSPs)

During transmission from sand fly to mammalian hosts, the parasite needs to acclimatize to the almost 10°C temperature upshift and the acidic pH (4.5–5.5) within the macrophage phagosomes. To overcome this challenge, the parasite induces the production of a plethora of leishmanial heat shock proteins (HSPs), which safeguards its proteins from heat-induced damages. Thus, parasite HSPs are key players during mammalian stage development of *Leishmania sp* (Figure 1).

### HSP100

HSP100 is an AAA+ casinolytic protease B (clpB) family protein, and through gene manipulation studies, it was found to play a non-canonical role in leishmanial virulence. HSP100-/- *L. donovani* and *L. major* parasites showed no growth impairment in axenic culture and had no sensitivity toward heat stress. However, both the species failed to thrive within mice models and failed to transform into amastigotes [117]. HSP100 is an amastigote-specific protein secreted from the parasite’s flagellar pockets via temperature-induced exosomal secretion pathway. HSP100 contributes to parasite virulence by playing a major role in exosomal trafficking pathway [118].

### HSP78

HSP78 is another clpB protease family member, which is an ATP-dependent amastigote-specific protein that assists in the management of heat and pH stress [119]. HSP78-/- *L. donovani* parasites are non-viable, and conditional knock out of HSP78 in *L. donovani* confirmed essentiality of the protein for promastigote growth. Moreover, partially depleted HSP78 *L. donovani* promastigotes showed impaired infectivity in macrophages and BALB/c mice. HSP78 plays a crucial role in suppressing pro-inflammatory responses and cytokines of macrophages, along with nitric oxide. In a pioneering study in this regard, Das *et al*. reported the regulatory role of HSP78 in the establishment of *Leishmania* infection in hamsters. ATP analogue, Ap5A, helped in identifying HSP78 as a potential chemotherapeutic target [120].

### Small HSPs

Small molecular weight HSPs (sHSPs) are mainly composed of a conserved α-crystallin domain (ACD), which folds in 7–8 stranded β-sandwich structure and exists in a dimer form. sHSPs are highly divergent compared to high molecular weight HSPs. sHSPs bind to a broad range of target proteins and function like holdase [121]. sHSPs mainly assist the ATP-dependent chaperons like HSP100 to fold the target proteins. HSP20, P23, and HSP23 are the well-characterized sHSPs from *Leishmania sp* [122–124]. HSP20 has been found to play an important role as a potential immunogenic antigen during canine leishmaniasis. However, its role as a protective DNA vaccine is still questionable. P23 and HSP23 from *L. braziliensis* function as HSP90 co-chaperones, and null mutants of P23 produced geldanamycin (HSP90 inhibitor)-sensitive parasites [125]. Further analysis of HSP23–/– parasites identified HSP23 as a heat-inducible chaperon, which is highly expressed in amastigote-like conditions and essential for amastigote stage differentiation. HSP23–/– parasites display enhanced sensitivity toward trivalent antimony Sb(III). HSP23 reportedly plays a potential role in resistance against trivalent antimony Sb(III) and metalloid-based anti-leishmanials, highlighting a potential connection of the sHSP with resistance generation against antimonial drugs [122].

### A2

A major rate-limiting step of *Leishmania* infection establishment inside host macrophage is the trans-differentiation of parasites from promastigote to amastigote. Formation of amastigote is crucial for the tolerance and survival of parasites inside the harsh phagosomal vacuoles. Amastigotes specifically possess some crucial virulence factors that are absent in the promastigote form of the disease. A2 is one such amastigote-specific protein that was first discovered in *L. donovani* by karyotype analysis. However, interestingly, A2 gene is mostly expressed in parasites causing VL but not CL. Moreover, antibody response against A2 gene was reported in human VL patients and infected dog sera but not in CL-infected individuals [126], indicating a prospective role of A2 in viscerelization of *Leishmania* parasites in mammalian organs [127]. A2 gene encodes for a total seven isotypes of proteins with molecular weights ranging from 45 to 100 kDa. A2 mRNA and protein expression are completely absent in promastigote but abundantly expressed in amastigote-like conditions, i.e. parasites cultured at 37°C and pH 4.5 [128] . A2 protein amino acid sequence shares unique repetitive stretch of 10 amino acids, which is homologous to that of S antigen of *Plasmodium falciparum* V1 strain. Interestingly, S antigen is another stage-specific virulence factor of *P. falciparum,* which is responsible for malaria infection in human host [128] . Antisense RNA-mediated A2-silenced *L. donovani* amastigotes determined that A2 deficiency severely hampered virulence and survival of amastigotes in both macrophages and BALB/c mice [129]. Besides, over-expressing A2 gene in *L. donovani* and *L. major* exhibited enhanced organ parasite burden in experimental animal model [130]. Immunization of mice with recombinant A2 protein or DNA vaccine significantly mounted protective immune response against *L. donovani* challenges [131]. This suggests that besides being a vital virulence factor, A2 possesses promising attributes to become a vaccine candidate.

### PTP1

Phosporlylation and dephosphorylation of proteins involved in significant biochemical pathways of *Leishmania sp*. play a crucial role during trans-differentiation from promastigotes to amastigotes. *Leishmania* genome database mining identified a group of protein phosphatases of parasites named protein tyrosine phosphatases (PTPs) [132]. Tyrosine phosphatases are involved in the removal of phosphate group from target protein, thereby regulating many essential life-cycle stages like cell cycle, differentiation, disease establishment, etc. Genetical manipulation studies confirmed that although LdPTP1 gene is not essential for the survival of *L. donovani* promastigotes, LdPTP1 null parasites are unable to persist in BALB/c mice. Due to the remarkable structural homology between human PTP1B and *L. inflantum* PTP1 active sites, in silico studies identified parasite PTP1 as a promising therapeutic target [133].

## Subversion of host defense machineries by *leishmania*

*Leishmania* parasites, like any other pathogen post entry, is readily taken up by host macrophages and other phagocytes. However, unlike most pathogens, *Leishmania* sp. tends to survive within the macrophages and have developed strategies to overcome the unfriendly intracellular environment designed for elimination of pathogens and foreign materials. Thus, leishmaniasis is a good infection model for studying host–parasite interaction. The parasite has viciously evolved various survival strategies to enter host macrophages and proliferate within by overriding the various defense weaponries of the host cells. Various reports suggest *Leishmania* sp. mediates modulation of various host processes like apoptosis and arsenals, like generation of ROS and RNS, and alters Th1/Th2 cytokine balance and TLR-mediated signaling mechanisms. *Leishmania* sp. parasites also possess the capability of modulating important host signaling pathways like MAPK and altering host miRNA pool to its favor. *Leishmania* sp.-secreted virulence factors interestingly either abrogate the functioning of host proteins/factors responsible for the activation of immune response or exploit host-negative regulatory proteins controlling immune functioning. In this section, we focus on few important strategies exploited by the parasite to alter the macrophage niche to a disease-conducive state.

## Suppression of reactive oxygen and nitrogen species generation

**Figure 2.**
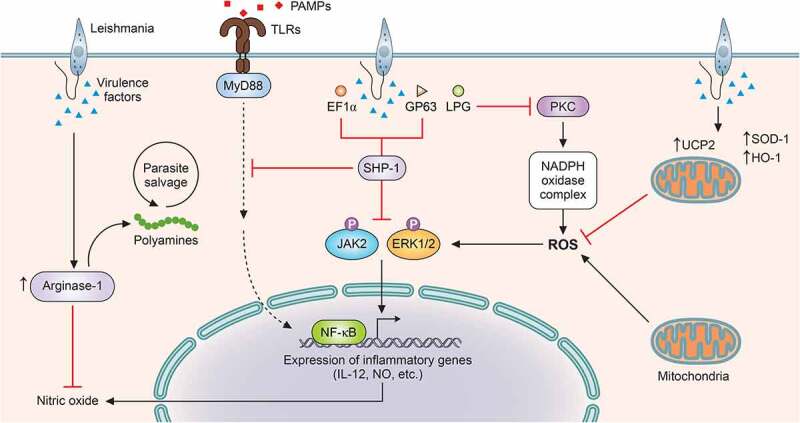
***Leishmania* suppresses ROS and RNS generation for successful survival within the host cell**. In order to tackle the burst of ROS and RNS on infection within the host, *Leishmania* has evolved various virulence factors as well as mechanisms. LPG of *Leishmania sp*. inhibits PKC activation, which is necessary for the formation of NADPH oxidase complex, thus, blocking ROS generation. For suppression of mitochondrial ROS generation, the parasite viciously exploits the mitochondrial membrane protein, UCP2. Apart from these strategies, the parasite has also been reported to upregulate host antioxidants like HO-1 and SOD1. In order to suppress NO production, the parasite exploits the host PTP like SHP-1 by virtue of virulence factors like EF-1α and GP63. SHP-1 blocks JNK and ERK activation, required for NO production. *Leishmania* also upregulates host arginase-1, which inhibits the harmful effects of NO. Apart from facilitating the parasite to overcome the effects of NO, host arginase also provides polyamines for parasite salvage (ROS: reactive oxygen species; RNS: reactive nitrogen species; LPG: lipopohosphoglycans; UCP2: uncoupling protein 2; HO-1: hemeoxygenase 1; SOD-1: superoxide dismutase-1; PTP: protein tyrosine phosphatase; SHP-1: Src homology 2 domain-containing protein tyrosine phosphatase 1; NO: nitric oxide; and EF-1α: elongation factor-1α). Image created through paid version of Biorender.

When a pathogen enters a host cell, one of the major challenges that it faces is the burst of ROS and RNS. Production of ROS and RNS by host cells to destroy invading pathogens or phagocytosed foreign materials are under tight regulation to ensure minimum collateral damage to the host. The process involves tightly controlled steps catalysed by various enzymes like NADPH oxidase 2 (NOX2) and nitric oxide synthase (NOS). The production of NO is catalysed by NOS by reacting with terminal nitrogen of the guanidium group of L-arginine. Early findings in this regard suggest that activated macrophages can inhibit *L. major* in an L-arginine-dependent manner [134]. This finding was later confirmed by studies indicating that usage of L-arginine analogue, L-N-monomethyl arginine, and inhibitors of NO pathway reversed the antileishmanial effect of IFN-γ or LPS-activated macrophages [135]. Their work also demonstrated that *in vivo* administration of L-arginine analogues in the *Leishmania-*resistant CBA mice rendered them susceptible to infection. In order to evade NO production, most leishmanial parasites exploit the host protein SHP-1 so as to interfere with JAK2, ERK1/2, and the transcription factors, AP-1 and NFκB. Moreover, SHP-1-deficient macrophages on infection with *L. donovani* could activate NO production [136], suggesting the importance of host SHP-1 for parasite survival. However, interestingly, reports by Spath et al. suggest that the intracellular survival of *L. major* was not dependent on SHP-1 (PMID: 18682252). *Leishmania*-mediated SHP-1 activation can be attributed to the parasite’s elongation factor-1α (EF-1α) [51]. The group, however, reported the results 16 h post infection and could not explain the activation of SHP-1 at early time points of infection, nor the shuttle mechanism of EF-1α from the phagolysosomes. Later studies by Gomez et al. suggested activation of host SHP-1 to be mediated by the metalloprotease of the parasite, gp63, known to traverse through lipid rafts to gain access to the host cytosol [137]. Another mechanism of downregulating NO production can be witnessed in *L. amazonensis,* which upregulates the expression of host arginase and polyamines [47]. Upregulation of arginase was reported in both susceptible and resistant laboratory mice on infection with *L. major,* which correlated with reduction in the expression of NOS2 [138,139]. Upregulation of host arginase also provides polyamines for parasite salvage and proliferation [139]. This can be justified by the finding that the arginase of the parasite, being an important enzyme for polyamine biosynthesis, is necessary for the survival of *L. donovani* promastigote but not the intracellular amastigote form [140]. This indicates a probable vicious strategy by the parasite of upregulating the host arginase not only to evade NO production but also to sustain its own biosynthetic processes. In this regard, another role of the parasite arginase can be witnessed in *L. amazonensis-*infected macrophages where the former led to the inhibition of miR-294 and miR-721, leading to increased NOS2 production [141] (Figure 2).

ROS or ROIs possess parasite killing capability, yet their effects can be considered transient and limited to host protection only during the early phases of infection [142]. However, for successful establishment of infection, the parasite must overcome the effects of ROS. *L. donovani* has been widely reported to inhibit ROS generation in infected macrophages [143,144]. *L. chagasi* infection has also been documented to register decreased superoxide production in murine and human macrophages [145]. *L. amazonensis* and *L. brasiliensis* have been reported to exploit the host anti-oxidant enzyme superoxide dismutase 1 (SOD1) as a survival strategy [146]. Similar to other intracellular pathogens like *Mycobacterium abscessus* and *Salmonella typhimurium* [147,148], various leishmanial species like *L. chagasi, L. pifanoi, and L. donovani* have also been reported to exploit another host antioxidant enzyme-hemeoxygenase 1 [149–151] (Figure 2). *L. pifanoi* infection induced HO-1 levels at a very early stage of infection, suggesting the necessity of cellular ROS evasion by the parasite for successful establishment of infection. NAD(P)H oxidase is the major contributor of cytosolic ROS in macrophages, and the different subunits of the enzyme complex are assembled on stimulation. Reports suggest inhibition of protein kinase C-mediated phosphorylation of p47 and its interaction with p67 by *L. donovani* amastigotes [152,153] (Figure 2), but strikingly, lipophosphoglycan (LPG) of *L. donovani* promastigotes blocks the assembly of the enzyme without involving p47 [154].

Besides the cytosolic ROS production by NADPH oxidase, mitochondria are a major contributor of intracellular ROS. The generation of mitochondrial ROS takes place due to premature leakage of electrons in the electron transport chain, which increase during physiological and pathological conditions [155,156]. Extensive studies implicate mitochondrial ROS generation with innate immunity and antipathogenic activity [156]. *L. donovani* has been reported to subvert the outburst of mitochondrial ROS by upregulating uncoupling protein 2 (UCP2), an inner mitochondrial membrane protein [143,144] (Figure 2). *Leishmania*-mediated upregulation of UCP2 has been reported to de-polarise mitochondrial membrane potential, eventually leading to altered electron transport and ROS formation [157].

Apart from exploiting host-negative regulatory proteins, *Leishmania* sp. themselves are armed with certain characteristics that provide resistance to the harmful effects of ROS and RNS. The layer of LPG, especially on the amastigotes form of the parasite, provides protection from ROS and RNS. It has been reported to delay the assembly of the NADPH oxidase 2 on the surface of the phagolysosomes [158]. The metalloprotease gp63 has also been implicated with the inhibition of various macrophage signaling requisite for the stimulation of NADPH oxidase and iNOS [158].

## Modulation of toll-like receptor-mediated signaling

**Figure 3.**
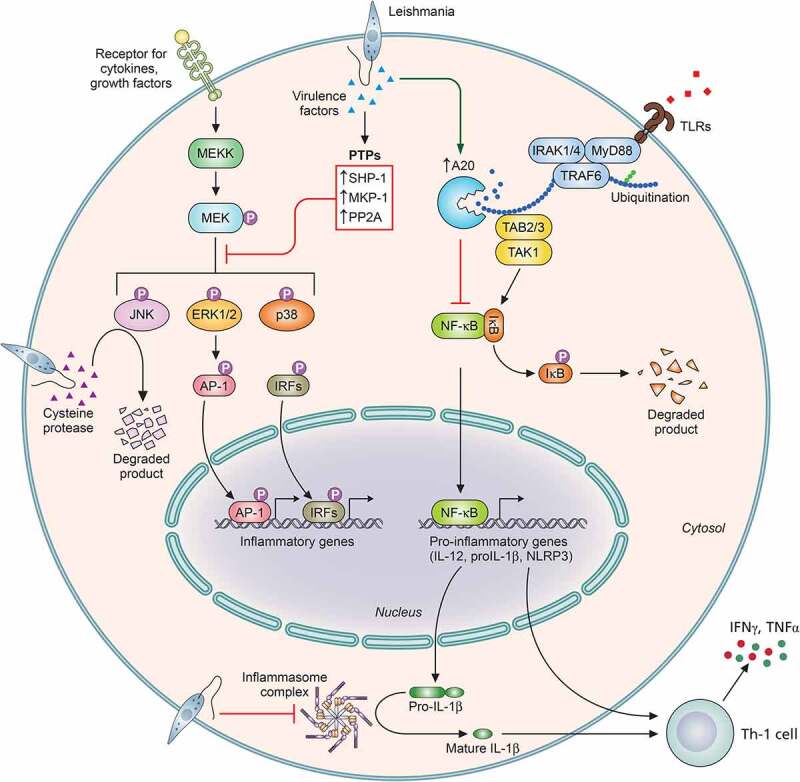
***Leishmania* overrides important MAPK and TLR signaling for successful survival**. Stimulation of TLRs triggers a signaling pathways that ultimately culminates in the activation and nuclear translocation of the transcription factor such as NFκB. Activation of MAPKs such as p38, JNK, and ERK1/2, through phosphorylation by upstream kinases, leads to the activation of the transcription factors-AP-1 and IRFs. Activation of NFκB, AP-1, and IRFs transcription factors activate the expression of pro-inflammatory genes and genes involved in host immune defense like IL-12, IL-1β, NLRP3, etc. In order to regulate TLR-mediated NFκB activation, the parasite upregulates the host deubiquitinases such as A20, which removes the necessary ubiquitination from TRAF6 and blocks its interaction with TAB/TAK complex. In order to block MAPK activation, the parasite exploits host PTPs like SHP-1, MKP-1, and PP2A. Furthermore, cysteine proteases of some species of *Leishmania* degrade ERK1/2 and JNK (NLRP3: Nod-like receptor protein 3; AP-1: activator protein 1; IRFs: interferon regulatory factors; SHP-1: Src homology 2 domain-containing protein tyrosine phosphatase 1; MKP-1: MAPK phosphatase 1; and PP2A: protein phosphatase 2A). Image created through paid version of Biorender.

TLRs, often considered the sentinels of the host, are capable of recognising pathogen-associated molecular patterns (PAMPs) by virtue of PRRs (pattern recognition receptors) similar to Nod-like receptors (NLRs), RIG-I-like receptors (RLRs), and cytosolic DNA sensors (CDs) [159]. TLRs behave as bridges between the innate and adaptive immunity of the host [159]. Post recognising PAMPs, TLRs induce a cascade of signaling pathways, leading to the nuclear localisation of NFκB and expression of antipathogenic products like pro-inflammatory cytokines and type 1 interferons [160] by macrophages and dendritic cells. However, macrophage-mediated phagocytosis of *Leishmania* is not associated with a burst of pro-inflammatory cytokines [161], and *Leishmania* infected macrophages have been reported to be unresponsive to LPS treatment [162], thus indicating the importance of TLR activation during infections by the parasite. TLRs play diverse roles from parasite clearance to aggravated pathology and are often species-specific in response to the various *Leishmania*-expressed ligands [163,164]. Extensive studies in this regard suggest the involvement of various leishmanial molecules in the activation of TLRs, specially TLR2, TLR4, and TLR9. TLR2-mediated recognition of LPG of *L. major, L. mexicana,* and *L. aethiopica* leads to increased ROS and NO production [165–167] and favors a protective immune response. Simultaneously, contrary reports exist suggesting TLR2 activation favoring persistence of *L. amazonensis* and *L. braziliensis* infection [168,169]. This differential response can be attributed to the differing thickness of the LPG coat of the mentioned species [169]. Association of TLRs and MyD88-mediated pathway during infection by *Leishmania* was first studied in 2002, reporting decreased IL-1α expression in MyD88^−/−^ mice upon infection by *L. major* [170]. The following study in C57BL/6 mice by Muraille et al. exhibited the importance of MyD88 in controlling cutaneous lesions by *Leishmania* along with enhanced IL-4 and reduced levels of IFN-γ and IL-12 [171]. Administering anti-IL4 antibodies led to an increase in the levels of IFN-γ and drove the cytokine balance toward disease resolving Th1 state [172]. The probable association of LPG with MyD88 and TLR2 was reported by de Veer et al., indicating the essentiality of MyD88 in the clearance of *L. major* infection [166]. The role of TLR2 in visceral leishmaniasis was reported in an *in vivo* model using TLR2 ligand, arabinosylated lipoarabinomannan, which registered enhanced NO and proinflammatory cytokine production. This led to a drastic decrease in the organ parasitic burden and a strong Th1 response [167]. TLR2 was also reported to be upregulated by 65 kDa and 98 kDa antigens from *L. donovani* amastigotes [173]. While studies by de Veere et al. and Debus et al. showed no effect of LPG and GPIL on TLR4 [166,172], later studies by Kropf et al. demonstrated the importance of TLR4-mediated iNOS activation on *L. major* clearance [174]. TLR4-mutated mice have been reported to heal cutaneous lesions caused by *L. major* [175]. *In vivo* studies with *L. chagasi* suggested infection-mediated upregulation of TLR-2 and TLR-4 along with increased expression of IL-17, TNF-α, and IFN-γ during early hours of infection [176]. *L. panamensis* infection in human primary macrophages also suggested upregulation of levels of TLR1, TLR2, TLR3, and TLR4. However, interestingly, the study associated TLR3 and TLR4 activity with increased TNF-α levels [177]. TLR4 has also been shown to sense glycospinghophospholipid antigen of the parasite and stimulate inflammatory signaling intracellularly, leading to parasite clearance [178]. In a recent study by Polari et al., *L. brasieliensis* infection was implicated with enhanced expression of TLR2 and TLR4, which triggered TNF-α and IL-10 production in monocytes isolated from CL patients [179]. TL4, on ligand binding, activates a downstream signaling cascade involving signalosome complex, which includes MyD88, TRAF6, IRAK1/4, and ubiquitin conjugating enzymes and eventually culminates in the activation of the NFκB pathway [180,181]. However, assemblage of the signalosome complex is interrupted during *Leishmania* infection [182]. Moreover, inhibition of the NFκB pathway is reported by upregulating the host deubiquitinase, A20 [144,183] (Figure 3). A20 deubiquitinates TRAF6 and hinders its association with TAK1, thus blocking TAK-1-mediated NFκB activation.

Apart from TLR2 and TLR4, TLR3 has also been reported to play an important role in *Leishmania* sp. infection. Flandin et al. in their study suggest involvement of TLR2 and TLR3 in the clearance of *L. donovani* via increased production of host NO and TNF-α [184]. The work further exhibits the importance of TLR3 in leishmanicidal activity of the IFN-γ-primed macrophages. Interestingly, stimulation of TLR3 during *L. guyanensis* infection induces hyperinflammatory response along with an increase in the parasitic burden and exacerbated disease symptoms [185]. This study associates the activation of TLR3 with the dsRNA of the endosymbiotic virus, LRV1, associated with the parasite. TLR9, on the other hand, is known to systematically resist *Leishmania* infection by inducing the secretion of IL-12, which ultimately leads to IFN-γ production by natural killer (NK) cells [185–187]. A therapeutic study against *L. major* suggested the probable involvement of CpG-TLR9 axis in parasite clearance. The study reports on the stimulation of proinflammatory cytokines, especially IL-12 mediated by TLR9 activation by CpG [186]. Liese et al. in their study found that *L. major*-infected TLR9-/- mice registered higher parasitic burden [188]. Another study reveals that *L. infantum* infection of dendritic cells led to TLR9-mediated production of IFNγ and increased levels of IL-12 [187].

Studies involving the roles of NLRs and CLRs in context of *Leishmania* sp. infection are currently being looked upon. Lima-Junior et al. suggested production of IL-1β via activation of NLRP3 inflammasomes to provide protection against infection to *L. amazonensis* and *L. brazilensis* by inducing NO production [189]. Interestingly, *L. donovani* exploits the host-negative regulatory proteins A20 and UCP2 to suppress the formation of NLRP3 inflammasomes (Figure 3). The study reveals that A20, a host deubiquitinase, blocks the NFκB activation, while UCP2, an inner mitochondrial protein, suppresses mitochondrial ROS generation in order to suppress the two activation steps of inflammasome formation [144]. However, strikingly different results have been reported in *L. major* infection, which aggravated post activation of NLRP3 inflammasomes by inducing IL-18-mediated Th2-based disease propagative response [190]. Reports concerning the role of CLRs in *Leishmania* infection suggest that stimulation of Dectin-1 and mannose receptors induces an antiparasitic oxidative stress, leading to clearance of *L. infantum* infection [191]. However, the same study reports an opposite role of the CLR, SIGN3, which promotes parasite resilience by inhibiting LTB4-IL1β axis. Moreover, another report suggests that *L. major* exploits Mincle to target ITAM signaling in order to block an adaptive immune response [192]. Despite these studies, a lot still remains to be explored for better understanding of the role of NLRs and CLRs in *Leishmania* infection.

## Modulation of host signaling pathways

For successful establishment of infection and proliferation within the host macrophage, *Leishmania sp*. subverts the various host defense machineries. This can be attributed to the capability of the parasite to alter and take over important host signaling pathways. This leads to the inhibition of host defense mechanisms and also subverts the various leishmanicidal functions, which are stimulated post activation of macrophage. *Leishmania* has developed strategies so as to either inhibit or suppress proteins that stimulate immune activation or upregulate negative regulatory proteins of the immune system and their functions.

Protein kinase C (PKC) family comprises of 10 serine/threonine kinases and were initially described as Ca^2+^ and phospholipid dependent [193]. PKC signaling has been implicated with regulation of macrophage activation and production of IFN-γ and TNF-α [194,195], eventually leading to the generation of NO and ROS [196]. Leishmanial LPG blocks PKC activity by binding to the regulatory domain of PKC, which possesses the diacylglycerol, Ca^2+^, and phospholipid binding sites [197]. However, interestingly, amastigote forms of *L. donovani* that are devoid of LPG can also inhibit PKC activity in monocytes [152], indicating the presence of other mechanisms exploited by the parasite to inhibit PKC activity. Olivier et al. reported that GIPLs from *L. major* amastigotes can inhibit PKC activity [198]. Another report suggests that *L. donovani* induces ceramide generation in murine macrophages to alter PKC activity as a survival strategy [199].

Apart from the PKC family, *Leishmania* infection exhibits negative regulation of Janus Kinase 2 (JAK2), a member of the Janus tyrosine kinase family. Activation of Janus Kinase signaling is implicated with important cell functioning like proliferation, migration, apoptosis, differentiation, and immune stimulation [200]. JAK signaling involves receptor binding of cytokines or growth factors, multimerization of the receptors, and activation by transphophorylation. These eventually result in the phosphorylation-mediated activation of signal transducer and activator of transcription (STAT), leading to nuclear translocation of the former and transcriptional activation or repression of various genes [200], including the previously discussed iNOS gene responsible for the production of NO. *L. donovani* promastigotes exploit the host SHP-1 to inhibit IFN-γ-induced JAK-2 phosphorylation and NO production [78]. Interestingly, IFN-γ-mediated STAT1α activation was also found to be abrogated upon infection by *L. donovani* in SHP-deficient macrophages, indicating the presence of other mechanisms employed by the vicious parasite to subvert STAT1 activation [136]. This is probably due to enhanced proteasomal degradation of STAT1 in *Leishmania*-infected macrophages [201]. Moreover, additional reports suggest attenuated levels of IFN-γ receptor alpha subunit [202] and increased transient expression of suppressor of cytokine signaling 3 (SOCS3), which have been known to negatively regulate IFN-γ signaling [203]. Further studies in this regard suggest *L. donovani* infection inhibits the nuclear translocation of IFN-γ-induced nuclear transport of STAT1α by blocking the interaction between STAT1α and importin-α5 [204].

Mitogen-activated protein kinases (MAPKs) belong to a group of serine/threonine kinase family including p38 MAPK, c-jun terminal kinase (JNK), extracellular signal-related kinases 1 and 2 (ERK1/2) and play a major accessory and effector role in host cells for production of NO and proinflammatory cytokines [205]. Activation of these pathways takes place post phosphorylation of Ser/Thr and Tyr residues present on their regulatory domain by MAP/ERK kinase (MEK) [206]. MEK is itself activated by upstream kinase-MEK kinase (MEKK) [207]. After activation, the kinases phosphorylate an array of intracellular proteins and transcription factors like NFκB, AP-1, and IRFs, triggering the stimulation of a diverse signaling cascade eventually regulating expression of various genes [208–210]. *Leishmania* as a survival strategy has been widely reported to modulate the alteration of MAPK (Figure 3). *L. donovani* has been reported to impair PMA-dependent activation of MAPK in infected macrophages to subvert the expression of c-FOS and ELK-1 [211]. *L. donovani* infection also enhanced the activity of host protein tyrosine phosphatase (PTP), SHP-1, which inhibits MAPK pathway [78,143] (Figure 3). These studies explain the findings that SHP-1-deficient macrophages exhibit JAK2 and ERK1/2 activation on treatment with IFN-γ in *L. donovani*-infected state [136]. *L. amazonensis* amastigotes, on the other hand, have been shown to block LPS-mediated activation of ERK1 [212]. Activation of other phosphatases like MKP1 and PP2A targeting ERK1/2 MAPK activation during *Leishmania* infection has also been reported [213]. Apart from phosphatases, *Leishmania* infection has been implicated with elevated endogenous ceramide in host macrophages [199]. Ceramide being an important intracellular lipid mediator regulates important cell functions like apoptosis and senescence [214]. Ghosh et al. in their study demonstrated intracellular ceramide-mediated dephosphorylation of ERK so as to dampen AP-1 and NFκB activation in *L. donovani*-infected macrophages [199]. Strikingly, *L. mexicana* amastigotes inhibit host ERK1/2 signaling not by phosphorylation but rather by enhancing their degradation. This is achieved by the parasite’s cysteine peptidases, which also have been reported to cause degradation of JNK [215] (Figure 3). p38 MAPK is also known to be inhibited during leishmanial infection [143,216], and its activation using anisomycin enhances parasite killing [217]. Ball *et al*. in their study reported the exploitation of the host inner mitochondrial membrane protein, UCP2, to suppress ROS-mediated activation of p38 MAPK [143]. An interesting study reported differential role of ERK and p38 MAPKs in regulating LPS-induced expression of iNOS and IL-12 [218]. p38 promoted the expression of IL-12 while ERK dampened LPS-induced IL-12 expression. The study further reports that synthetic *Leishmania* LPG targets ERK MAPK to subvert the production of IL-12 as a survival strategy. However, a contradictory report suggests that *Leishmania* LPG promotes pro-inflammatory and endotoxin-like response and stimulates the production of IL-12 and NO. This is achieved by LPG-mediated activation of p38 and ERK MAPK, leading to activation of AP-1 [219]. Apart from the above mechanisms exploited by the parasite, Halle et al. demonstrated that the leishmanial metalloprotease, GP63, also inactivates p38 MAPK, possibly by degrading the upstream adaptor TAB1 [220].

### Host cell apoptosis and leishmania

Apoptosis is a natural phenomenon in multicellular organisms whereby a host cell undergoes programmed cell death. It is an important component of various cell functioning like normal turnover of cells, hormone-mediated atrophy, immune system functioning, and development of embryo [221]. Since intracellular pathogens tend to survive within the cellular niche of the host, apoptosis serves as the ultimate resort for infected cells so as to completely eliminate the former by destruction of its own self. Hence, most intracellular pathogens have developed strategies to ensure inhibition of apoptosis of host cell as a surviving strategy.

The anti-apoptotic host protein myeloid cell leukemia-1 (MCL-1), which is a member of the Bcl-2 family, has been implicated with various intracellular infections [222,223]. Reports suggest enhanced RNA and protein levels of MCL-1 during infection by virulent but not attenuated strains of *Mycobacterium tuberculosis* [222]. Similarly, *Leishmania* infection has also been associated with increased MCL-1 expression. In a recent study, Das et al. demonstrate that unmethylated CpG motifs in *L. donovani* DNA regulate TLR9-mediated delay in the programmed cell death of the host macrophage [224]. Significant upregulation of MCL-1 during infection by *L. donovani* was also reported by another group, which further suggests infection-induced MCL-1 to be localised in the host mitochondria. The study reports that parasite-induced MCL-1 associates with Bcl-2 homologous antagonist/killer (BAK) and inhibits its homo-dimerization and eventual release of mitochondrial membrane cytochrome c [225]. Recently, a significant level of Bcl-2 was reported in the peripheral blood of VL patients. The study further exhibited that Bcl-2 inhibition led to increased NO-mediated parasite clearance [226]. Furthermore, the parasite has also been reported to indirectly inhibit host apoptosis by improving the stability of PTPs in a SOCS-dependent manner. PTPs in turn inhibit the activation of the caspase cascade necessary for triggering apoptosis of host cells [227].

Further, the parasite also exploits the multifaceted regulator, AKT signaling pathway, to inhibit host defense mechanisms like production of inflammatory cytokine and host cell apoptosis. Phosphoinositide-3 kinases (PI3K) on interaction with membrane receptors phosphorylate inositol phospholipid producing the secondary messenger, inositol-3,4,5-triphosphate, which recruits and activates AKT (protein kinase B). A recent study by Gupta et al. suggests that *L. donovani* infection triggers AKT activation to inhibit GSK-3β. Inhibition of GSK-3β leads to the activation of the anti-apoptotic, β-catenin, and concomitant inactivation of the pro-apoptotic Forkhead box protein O1 (FOXO-1) [228]. However, the surface molecule of the macrophage with which the parasite interacts triggering the PI3K/AKT pathway is still not known. Interestingly, reports in Schwann cells suggest that *L. major* does not trigger the PI3K/AKT pathway [229], indicating the presence of other strategies exploited by this parasite.

## Modulation of host non-coding RNAs as a survival strategy

MicroRNAs (miRNAs) are 18–22 nucleotide long non-coding RNAs (ncRNAs) that post-transcriptionally regulate the turnover, translation, and expression of a specific group or cluster of mRNAs. Monocistronic or polycistronic (miRNA cluster) form of miRNAs can get transcribed from host exon or intron sequences in a RNA polymerase II-dependent manner. miRNAs can be encoded either from their own promoters or can utilize promoters regulating other mRNA expression (if miRNAs are intragenic). After transcription, miRNA obtains a folded double-stranded hairpin-like conformation known as pri-miRNA and gets processed by class 2 RNase III DROSHA and DGCR8 to form pre-miRNA inside the nucleus [230]. Pre-miRNAs get exported from nucleus to cytosol in Exportin 5- Ran GTPase-dependent fashion and processed into miRNA duplex by Dicer1-transactivation response element RNA-binding protein complex (Dicer1-TRBP) [231]. The functional and mature miRNA strand of this duplex then couples with RISC-Argonaut 2 (AGO 2) to form micro-ribonucleoprotein (miRNP) complex [232]. miRNP interacts with the “seed” sequence of the 3’ untranslated region (UTR) of target mRNA with partial or complete sequence complementarity to accomplish their translational suppression or degradation, respectively [233] (Table 2).

**Table 2.** List of host non-coding RNAs modulated by *Leishmania* sp.

Name of non-coding RNAs	Target genes	Possible virulence factor involved	Function during Leishmanial infection
miR-9	PPAR-δ	… … .	Suppresses M1 function [234]
miR-146a-5p	TRAF6, IRAK1	… … .	Promotes M2 polarization by targeting TLR4 pathway [235,256]
miR-181a	C/EBP-α, KLF6	… … .	Promotes M2 polarization and suppresses M1 function [236]
miR-124	C/EBP-α	… … .	Suppresses inflammatory response [237]
let-7c	C/EBP-δ	… … .	Suppresses inflammatory response [238]
miR-210	NFκB	… … …	Suppresses M1 polarization [239]
miR-130a/b	PPAR-γ	… … .	Promotes M1 and inflammatory response [240]
miR-26a	PPAR-γ	… … .	Promotes M1 and inflammatory response [241]
miR-21	SMAD7, PU.1	… … .	Targets JAK-STAT signaling and suppresses inflammation [250]
miR-720	GATA3	… … .	Promotes M1 and inflammatory response [242]
miR-511	TLR4	… … .	Promotes anti-inflammation and survival of parasites [25]
miR-122	Cationic amino acid transporter 1 (CAT1)	gp63, host c-Myc	Promotes parasite survival via lowering serum cholesterol [257,260]
miR-294-3p	NOS2, TNFα	leishmanial Arginase	Promotes parasite survival via suppressing NO and ROS production [260,262]
miR-30e	NOS2	… … …	Promotes parasite survival via suppressing NO production [260]
miR-302	NOS2	… … …	Promotes parasite survival via suppressing NO production [262]
miR-721	NOS2	… … …	Promotes parasite survival via suppressing NO production [260]
miR-210	p-50 subunit of NFκB	… … …	Promotes M2 polarization and suppresses NF-κB pathway-mediated activation of IL12a and TNFα [266]
miR-361-5p	TNFα, Granzyme	… … .	Prevents TNFR-mediated clearance of parasites [267,268]
miR-193b	TNFα	… … .	Prevents TNFR-mediated clearance of parasites [267]
miR-671	TNFα	… … .	Prevents TNFR and CD40-mediated clearance of parasites [267]
miR-548d-3p	TNFα, Granzyme	… … .	Prevents TNFR-mediated clearance of parasites [267]
miR-346	TAP-1, RFX1 and BCAP31	… … .	Subverts MHC I/II-mediated antigen presentation [270]
miR-466i	MyD88	… … .	Subsides TLR4 pathway and upregulate IL10 expression [271]
miR-30a	Beclin (BECN1)	… … .	Prevents autophagy activation for parasite survival [274]
miR-574	STAT4	… … .	Downregulates IFN-γ response from CD4 + T cells [275]
miR-6994-5p	STAT1	… … .	Downregulates IFN-γ response from CD4 + T cells [275]
miRNA-6994-5p	IFN-γ	… … .	Downregulates IFN-γ response from CD4 + T cells [275]
miR-5128	IFN-γ	… … .	Downregulates IFN-γ response from CD4 + T cells [275]
miR-7093-3p	IL12a	… … …	Downregulates IL12 signaling cascade of Th1 cells [275]
miR-574-5p	NFAT	… … …	Downregulates IL12 signaling cascade of Th1 cells [275]
miR-7235	ZAP70	… … …	Downregulates IL12 signaling cascade of Th1 cells [275]
let-7a	SOCS4	… … …	Downregulates IL12 signaling cascade of Th1 cells [250]
miR-155	Arginase 2	… … …	Promotes antigen presentation and parasite clearance via DC [261]
Alu RNA	Not known yet	gp63	Prevents infection establishment in phagosomal vacuole [276]
Signal recognition particle RNA	Not known yet	gp63	Prevents infection establishment in phagosomal vacuole [276]
B1 RNA	Not known yet	gp63	Prevents infection establishment in phagosomal vacuole [276]

After infecting the mammalian host, *Leishmania* sp. enters into the phagocytic cells of the hematopoietic lineage like neutrophils, macrophages, and dendritic cells (DC) where it trans-differentiates from promastigote to amastigote form [243]. In order to downregulate the activation of initial inflammation and establish a successful immune-suppressive microenvironment, a complex host–parasite interaction prevails, which includes alteration of immune regulatory miRNA expression profile [244]. Like T_helper_ (T_h_) cells, macrophages are also plastic in nature, which can shuffle between pro-inflammatory “classically activated” (M1) form and anti-inflammatory “alternatively activated” form (M2) depending upon the environmental cues present. Macrophages stimulated with endotoxins like lipopolysaccharide (LPS) and T_h_1 cytokines like interferon-γ (IFN-γ) differentiate into M1 type. On the contrary, stimulation with Vitamin D3, macrophage colony stimulating factor (M-CSF), T_h_2 cytokines like interleukin 4 (IL4), and interleukin 13 (IL13) activates M2 type of macrophages [245]. M1 macrophages are characterized by enhanced antigen-presenting properties, transcription factors like NF-κB, PU.1, and capacity to secrete pro-inflammatory cytokines like IL1β, IL6, IL12, tumor necrosis factor-α (TNFα), etc. The M1 cytokines engage T_h_1-adaptive arm, which cross-induces parasite clearance from macrophages by triggering nitric oxide and oxidative burst [246]. On the contrary, *L. donovani* triggers M2 polarization via activating mTOR, PPAR-γ, and CD163 [247–249]. M2 macrophages activate the T_h_2 arm by IL10, TGF-β, IL-4, etc. These T_h_2 cells eventually assist in the initiation of polyamine biosynthesis pathways in infected macrophages in an arginase 1-dependent manner, which favors parasite growth [246].

In the last few years, small RNA profiling of various macrophages infected with *Leishmania sp*. uncovered that *L. donovani, L. major, L. amazonensis*, and *L. infantum* parasites can potentially alter macrophage immuno-miRs expression to favor their own proliferation and survival within macrophages [250–252]. Li *et al.* reported that miR-9, miR-146a/b, miR-181a, miR-124, let-7c, and miR-210 are responsible for promoting M2 phenotype, whereas miR-130a/b, miR-125a/b, miR26a, miR-21, and miR-720 promote M1 phenotype [253]. Small RNA profiling of *L. amazonensis*-infected murine macrophages revealed a mixed M1- and M2-type response during infection [254]. Geraci *et al.* reported enrichment of miR-21 and let-7a, which are known to target JAK-STAT signaling mediators SMAD7 and SOCS4, respectively, by small RNA profiling of human monocyte-derived macrophages (human MDM) and DC cells infected with *L. donovani*. They also found enrichment of miR-511 and miR-21, which target TLR4 pathway and PU.1 transcription factor, respectively [250]. Nimsarkar *et al.* consolidated chemical systems biology and synthetic biology approach to uncover unique juxtacellular export of miR-146a-like elements from *L. major* to macrophage cytoplasm, which target SMAD4, the negative feedback regulator of TGF-β signaling cascade, and promote M2 profile [255]. Later Das *et al.* reported enrichment of M2 polarizing miRNAs like miR-146a, miR-181a, and miR-125a and downregulation of M1 polarizing miR-26a in *L. donovani*-infected mouse BMDMs. Here authors reported that miR-146a promotes M2 polarization by targeting TRAF6- and IRAK1-mediated activation of NFκB module and IL12-*i*NOS axis [256]. These findings indicate a possible correlation of *Leishmania* infection-mediated alteration of macrophage plasticity and miRNAs.

Besides inflammatory genes, parasites can modulate global landscape of host metabolism by directly targeting miRNA biogenesis with different virulence factors. *L. donovani* delivers zinc metalloprotease gp63 in host hepatocyte cytosol via exosomes, which cleaves miRNA processing enzyme Dicer1. Degradation of Dicer1 leads into inhibition of miRNP-mediated biogenesis of miR-122 in hepatocytes and contributes to lowering of serum cholesterol level and facilitates infection progression in liver [257]. Besides being a serum biomarker for canine VL, miR-122 can become a potential therapeutic target for lowering liver parasite burden [258]. *Leishmania sp*. can extraordinarily exploit host transcription machinery in order to regulate cellular abundance of miRNAs. In human MDMs, *L. donovani* exploits host c-Myc as a proxy virulence factor in order to induce overall miRNA suppression. Therefore, host c-Myc serves as a novel proxy host protein harnessed by *L. donovani* for its survival [259]. In addition to gp63, parasite arginase can utilize host L-arginine source and prevent inflammatory nitric oxide generation. However, along with its own arginase 1, parasites harness host macrophage arginase as well via miR-122-mediated repression of cationic amino acid transporter 1 (CAT1) [260]. *L. donovani* infected macrophages, and DCs display lower burden of miR-155. miR-155 prevents parasite survival by repressing arginase 2 in DCs and allows activation of T cells; hence, by downregulating miR-155, the parasites promote their own survival in DCs [261]. In order to subvert nitric oxide-mediated clearance within macrophages, *L. amazonensis* reportedly upregulates miR-294-3p, miR-30e, miR-302, and miR-721 levels, which target NOS2 mRNA and prevent NO generation [260,262]. Besides nitric oxide, miR-294-3p is also involved in downregulating TNFα transcript, which helps the parasites to evade ROS-mediated toxicity [262].

Parasites attempt to diminish the chemokine response of macrophages by means of certain miRNAs. For example, *L. major* infection causes significant downregulation of CCR2, CCL5, CXCL10, etc. via up regulating let-7a, miR-25, miR-26a, miR-132, miR-140, miR-146a, and miR-155 in human macrophages [263,264]. Likewise, *L. donovani* and *L. amazonensis* infection induce hypoxic environment in the macrophages via activation of hypoxia-inducible factor 1α (HIF-1α) [265,266]. In *L. donovani*-infected macrophages, HIF-1α activates miR-210 expression, which suppresses NF-κB pathway-mediated activation of IL12a and TNFα by targeting p-50 subunit [266]. These findings postulated an interesting survival strategy of *Leishmania* parasites via hypoxia-mediated miRNAs activation. miR-21 is another major regulator of IL12a in *L. donovani*-infected murine macrophages and DCs . In pentavalent antimonial-resistant localized cutaneous leishmaniasis (LCL) patients, *L. braziliensis* infection stimulates miR-361-5p, miR-193b, and miR-671 expressions to prevent TNFα, CD40, and TNFR-mediated parasite clearance from cutaneous macrophages [267]. miR-361-3p and miR-548d-3p serve as poor prognosis markers of active CL. During *L. (viannia) braziliensis* infection, these miRNAs mainly target granzyme (GZMB) and TNF pathway activation to support parasite survival [267,268].

In addition to human and murine VL models, in canine VL, *L. infantum* has been found to differentially modulate miR-150, miR-451, miR-192, miR-194, and miR-371 titers in peripheral blood monocytes (PBMCs). These miRNAs are reportedly involved in downregulating inflammatory response of macrophages by targeting TNF-α, CD80, and IFN-γ [269]. Macrophages are one of the professional antigen-presenting cells, which bridge pathogen infection with the adaptive arm of the immune response via class I and II major histocompatibility complexes (MHCs) [269]. *L. infantum* can strategically overcome MHC I/II-mediated antigen presentation by targeting TAP-1, RFX1, and BCAP31 via miR-346 in human U937 and THP-1 cell lines [270]. Another major APC is DCs, where TLR4 pathways play crucial roles for downstream activation of inflammation upon parasite infection. In SAG-resistant *L. donovani* infection, enrichment of miR-466i reportedly targets MyD88 to subside TLR4 pathway and upregulate IL10 expression [271]. Besides this, *L. amazonensis* targets TLR-dependent activation of NOS2 through enriching let-7e levels. Muxel *et al.* showed that TLR pathway mediators like *Traf*6, *Ppara, Mapk*8*ip*3/*Jip*3, *Tnfpaip*3, *Map*2*k*4, *Tbk*1, and *Tnf* are upregulated upon inhibition of let-7e, indicating that parasite exploits the miRNAs for subsiding TLR-pathway-mediated clearance [272]. TLR mediators limit parasite infection not only by activating inflammation but also autophagy activation. C57BL/6 mice lacking endosomal TLRs like TLR3, TLR7, and TLR9 are highly susceptible to *L. major* infection due to impaired autophagy activation [273]. In addition, *L. donovani* can negatively regulate Beclin1 (BECN1) via miR-30a in human MDMs and THP-1 macrophages [274].

Small RNA profiling revealed that *L. donovani* can differentially regulate expressions of miRNA profile that are involved in the plasticity of CD4+ T cells. Among the pathways affected by *L. donovani* are Notch, JAK-STAT, IFN-γ, and MAPK. Kumar *et al*. reported that *L. donovani* prevents IFN-γ secretion from T_h_1 response by upregulating miR-7a-1-3p, miR-690, miR-7017-5p, miR-574-5p, and miR-7235-5p. miR-574 and miR-6994-5p target STAT4 and STAT1, respectively, and miRNA-6994-5p and miR-5128 target IFN-γ genes, which ultimately downregulate IFN-γ signaling cascade in CD4+ T cells during *L. donovani* infection. Additionally, the authors reported that by upregulating miR-7093-3p, miR-5128, miR-574-5p, and miR-7235, *L. donovani* targets IL-12 R, NF-AT, and ZAP70, which together hamper IL12 signaling cascade of Th1 cells [275].

Apart from miRNAs, recent reports suggested that *L. donovani* infection can negatively modulate small ncRNAs (Alu RNA, B1 RNA, and signal recognition particle RNA) for successful establishment of infection in phagosomal vacuoles of macrophages. These ncRNAs have signature B-box element at their promoter regions, which require recruitment of RNA pol III transcription factor TFIIIC for transcription. *L. donovani* gp63-based activation of thrombin receptor PAR1 increases the cytosolic Ca^2+^ levels and hence activates Ca^2+^-dependent calpain protease, which degrades TFIIIC [276]. In addition to *L. donovani*, computational prediction identified a role of ncRNAs in gene expression modulation during *L. amazonensis, L. braziliensis, L. infantum*, and *L. major* infection [277].

## Role of host epigenetic factors in *leishmania* infection progression

Recent studies highlight subversion of host macrophage immune response by *Leishmania* largely by modulating histone dynamics of many major inflammatory genes by different histone modifiers known as “writer” proteins. Parmer *et al.* was the first to reveal an interesting correlation between histone methylation and macrophage polarization during VL. They reported that *L. donovani* represses TNFα expression by Smyd2-mediated H3K36 dimethylation of TNFα promoter and *i*NOS gene expression by Ezh2-mediated H3K27 trimethyation of NOS2 promoter in J774 macrophages. Moreover, *L. donovani* induces IL10 expression by Ash11-mediated H3K4 trimethylation of IL10 promoter [278]. *L. amazonensis* also exploits host histone modification system to survive inside macrophages. Histones deacetylases (HDACs) form complexes with p50/p50-dimer that remove H3K9Ac acetylation and CBP/p300 from *i*NOS promoter and suppress its expression. Thus, by upregulating HDAC1, *L. amazonensis* induces hyporeactive stage in infected patients [279]. Similarly, in *L. donovani*-infected THP-1 cells, HDAC1 was enriched, which subsequently removes H3K27 acetylation from macrophage defense gene promoters. Thus, Roy *et al.* proposed HDAC1 inhibitors as potential anti-leishmanial agents [280]. Hijacking host histone H3 modification is a strategy of *Leishmania* parasites to target inflammasome pathway as well [281]. Histone H3K9/14 hypoacetylation and H3K4 hypo-trimethylation induced by *L. amazonensis* help the parasite to subvert NLRP3 and NF-κB activation [282]. Das *et al.* identified a unique mode of miRNA regulation via super enhancer elements (SE) formation at miRNA promoters in *L. donovani*-infected macrophages and BALB/c mice. SE elements are clusters of multiple enhancers, longer in length and occupied by extraordinary writer proteins like bromodomain 4 (BRD4), p300, RNA pol II, etc. The authors described that BRD4 plays a crucial role in *L. donovani* infection-induced SE element formation at upstream of miR146a-5p promoter, which drives M2 polarization of macrophages [256].

## Approaches in *leishmania* diagnosis and the virulence factors

Diagnosis of leishmaniasis can be done either directly by observing the parasite in the infected patients’ samples through microscopy or by indirect methods of detecting leishmanial genetic materials and proteins such as virulence factor. The direct method of detecting *Leishmania* parasites by the microscopic visualization of amastigotes in the infected tissue is the most rational way of diagnosis, and it is still accepted as a gold standard test in many endemic regions worldwide. Interestingly, newer studies suggest various leishmanial virulence factors as antigens to possess diagnostic potential in terms of antigens or antibodies in response to the factors. Antigens or antibodies are used in diagnosis mainly in the ELISA and immunochromatographic format.

The diagnostic performance of the test depends largely on the protein used in the assay. The use of ELISA with crude soluble proteins isolated from *Leishmania* promastigotes traditionally show varying sensitivity and specificity depending on the protein used [283–286]. Cross-reactivity of total crude antigens with other similar infections like trypanosomiasis, toxoplasmosis, and tuberculosis has been observed in many cases. As a result, search for a specific leishmanial protein that has high sensitivity and no cross-reactions with other diseases is still in progress. In one of the studies, *L. donovani* proteins of different molecular weights such as 31, 34, 36, 45, 51, 63, 72, 91, and 97 kDa were purified and evaluated in a serological ELISA. It was observed that 34 and 51 kDa proteins showed 100% sensitivity and no cross-reactivity with other diseases [287]. Advancement in molecular biology led to the discovery of more defined recombinant proteins that were easier in purification with no batch-to-batch variation, unlike crude proteins. A number of recombinant *Leishmania* proteins have been cloned and studied for serological diagnosis, such as rK9, rK26, rKRP42, rKE16, rA2, rKDDR, and rK39. Among all, rK39, a kinesin-related protein of *L. chagasi,* is reputed as the best recombinant protein for VL diagnosis, especially in the context of the Indian subcontinent. Roughly, 88.6–99% sensitivity and 81–98.2% specificity have been observed for the diagnosis of VL caused by *L. infantum* and *L. donovani*. Using Indian sera, a recombinant protein cysteine protease C (CPC) proved to be a good diagnostic candidate with 98.15% sensitivity as compared to 92.59% and 96.29% with glycoprotein 63 (GP63) and elongation factor 1-α (EF1- α), respectively. In a similar study, CPC with urine showed a sensitivity of 96% than 90% and 84% for GP63 and EF1- α, respectively [288]. Since the antibodies continue to be present in the blood after VL treatment, most of the leishmanial proteins cannot be used as a test of cure. However, a recombinant protein, *L. donovani*–otubain cysteine peptidase (Ld-OCP), showed complete clearance of antibodies in follow-up patients after 6 months of treatment [116]. Several proteins such as rHSP83, LiHyE, HSP70, HSP83.1, rLb8E, and rLb6H have been tested in CL and MCL diagnosis, and promising preliminary results were reported in ELISA [286].

To overcome the limitations of the invasive parasitological method and laboratory-based serological tests, the point-of-care test has been developed for simple and field-adaptable diagnosis. The World Health Organization (WHO) has approved the ASSURED (affordable, sensitive, specific, user-friendly, rapid and robust, equipment-free, and deliverable to end-users) criteria of point-of-care tests used primarily for infectious diseases diagnosis. Immunochromatographic test (ICT) has proved to be a benchmark in the field diagnosis of NTDs, including leishmaniasis due to its high performance, low cost, simplicity, and promptness. ICT in dipstick format with *L. donovani* antigen, LAg demonstrated 100% specificity with Indian and Brazilian VL sera, as well as Indian VL urine [284,285]. Further, in a multicentric study, serum-based dipsticks showed 97.10% sensitivity and 93.44% specificity in six countries, India, Sri Lanka, Nepal, Ethiopia, Brazil, and Spain [289]. Similar to ELISA, leishmanial proteins were also used in ICT to detect antibodies present in the infected samples in a lateral flow assay (LFA) format. An LFA is a rapid screening test where protein is coated on the nitrocellulose membrane. The binding of coated protein and sample antibodies can be seen directly as a color band due to tracer molecules like colloidal gold. Using crude antigen, LAg in LFA exhibited sensitivities of 96.49% and 95.12% and specificities of 95% and 96.36% with Indian VL sera and urine, respectively, infected with *L. donovani*. In a similar study, 88.57% sensitivity and 94.73% specificity were monitored with the Brazilian sera infected with *L. infantum* [290]. Among recombinant proteins, rK39-ICT has certainly been the widely used rapid test for VL diagnosis in the last decade. However, its performance shows drastic variations among different geographical locations of VL endemicity. Sensitivity rates of rK39-ICT performed in the Indian subcontinent were determined to be 97%, whereas in Brazil and East Africa, sensitivities go down to 85% and 67%, respectively [290,291]. The disparity in rK39-ICT performance initiated the development of numerous proteins for ICT that were isolated from regional *L. donovani* species, such as rK9, rK26, rK28, rKE16, and rKRP-42. Most of these recombinant proteins are kinesin-related, and their performance varies in VL diagnosis. Among non-kinesin proteins, Ld-OCP showed 100% sensitivity as compared to rK39-ICT, which showed 83.33% and 64.10% sensitivity with the Indian and Brazilian sera, respectively [116]. Interestingly, as a test of cure, Ld-OCP did not recognize antibodies in *L. donovani*-infected patients after 6 months of treatment; in contrast, rK39-ICT still reacted with 86.66% follow-up serum samples [116].

In spite of the knowledge gathered on different leishmanial virulence factors and their use in drug target identification, diagnosis is also an area where these factors have been used directly or indirectly.

## Limitations of available antileishmanial therapies

Therapy against leishmaniasis are limited and do not meet the satisfactory standards, and most of the drugs used against leishmaniasis were repurposed from other diseases [292]. Although patients respond to most of the available drugs by exhibiting apparent cure within 2 weeks, significant cases of relapse of leishmaniasis have been reported within 6 months of treatment. However, with the advancement in our knowledge of important virulence factors and enzymatic pathways of *Leishmania*, additional selective drugs are being proposed. Synthetic and natural compounds targeting important leishmanial enzymes/factors like topoisomerases [293], Hsp78 [120], and Aurora kinase [294] are being studied. Moreover, the knowledge of the various subversion strategies of the parasite to evade the host immune defense has led to the advent of various natural immunomodulators as futuristic antileishmanial therapy.

Pentavalent antimonials are the first-line treatment used extensively worldwide against both VL and CL. Among them, Sodium Stibogluconate and Meglumine Antimonate are clinically used for over seven decades. In spite of the extensive usage, the exact antileishmanial mechanism of pentavalent antimonials is not well elucidated even today. However, like most other drugs, antimonial therapy is also associated with various toxicities like cardiotoxicity, hepatotoxicity, and other adverse effects like pancreatitis and arthealgia [295].

The antimonial drugs showing poor oral absorption has to be administered intravenously or intramuscularly at a dose of 20 mg/kg for 20–30 days [292]. Apart from this, the high cost of the drugs limited their use in developing countries. In order to tackle the cost, a cheaper generic version Sodium Antimony Gluconate (SAG) was developed by Albert David in India [296]. However, in the last two decades with the emergence of resistance among patients, the drug has lost its efficacy. The emergence of resistance may be attributed to long drug exposure either due to elongated drug regimen or the long half-life of the drug. In order to overcome this problem, several liposomal formulations of the drug are being tried and are showing promising results [297]. Recent advances in improving the efficacy of antimonials include combination therapies [298].

Due to the dearth in the knowledge of validated drug targets, most of the other antileishmanial drugs available were a result of drug repurposing. The widely used antifungal drug amphotericin B (AmpB) is one of the first drugs repurposed in this regard. The antifungal drug Amphotericin B is a polyene macrolide, which is used in the deoxycholate form as a frontline antileishmanial therapy in patients with antimony-resistant parasites [292]. Since amphotericin B binds with sterols of host cell walls, it inhibits the interaction with promastigotes, thus hampering the internalisation of the parasite [299]. Later studies by Chattopadhyay et al. suggested a novel antileishmanial mechanism of AmpB. They reported AmpB-mediated targeting of the sterols like ergosterols present in the cell wall of the parasite leading to lysis of the cell [300]. AmpB with an antileishmanial efficacy of >95% has been reported to be highly effective against VL [301] and Indian PKDL [302]. However, AmpB infusions in patients were associated with adverse toxicities like renal complications and hypokalemia [303]. Liposomal formulations of AmpB like Ambisome, Fungisome [304], amphotericin B lipid complex, and amphotericin B cholesterol dispersion exhibited better clinical results with improved drug stability and targeted delivery. Ambisome has already been approved by US FDA and has been registered for use as antileishmanial drug in India, Egypt, and Brazil [305]. However, cholesterol is an important constituent of the formulation, and at the same time, it is known to facilitate the internalisation of the parasite in VL. In order to overcome this hurdle, a modified lipid formulation, Kalsome^TM^10, with AmpB intercalated with sterols and phosphatidylcholine was introduced. Kalsome^TM^10 exhibited improved parasite clearance and minimum-associated toxicities [306,307]. Apart from suppressing disease-conducive environment in the host, Kalsome^TM^10 also induced apoptosis-like cell death in the parasite [308], proving it to be a better alternative to Ambisome or other liposomal formulations of AmpB.

Miltefosine (hexadecyl phosphocholine) was the first drug to be registered in India, which was orally administered [309]. Miltefosine at a dose of 50–100 mg for 28 days exhibited around 94% cure [310]. Miltefosine is known to impair the synthesis of cell surface components of the parasite eventually, leading to programmed cell death [311,312]. Miltefosine also possesses immunomodulatory effects on macrophages and has been reported to enhance phagocytosis [313]. A study by Gupta et al. reports that miltefosine treatment in infected hamsters shifted the cytokine balance to the disease, resolving Th1 type by inducing IL-12, TNF-α, IFN-γ, and iNOS production, simultaneously decreasing the production of anti-inflammatory cytokines [314]. Similar potency of shifting the Th1/Th2 cytokine balance towards Th1 by miltefosine was also reported in Indian PKDL patients [315]. However, like the other two frontline drugs, miltefosine has limited usage due to its high cost and associated toxicities like gastrotoxicity, heptaotoxicity, and nephrotoxicity [309]. Moreover, miltefosine is teratogenic in nature; its usage by pregnant women is limited as it increases the chances of contraception and needs monitoring during treatment for at least 4 months after treatment. Furthermore, the long half-life of the drug increases the probability of drug resistance [292].

## Advent of immunomodulators: Futuristic antileishmanial therapy?

In order to tackle the limitations of available drugs, extensive research focused on identifying leishmanial virulence factors as putative drug targets are being carried out. The importance of HSPs, DUBs, and various kinases in the virulence and pathogenicity of the parasite marks them as potent antileishmanial drug targets. Apart from the leishmanial virulence factors, the strategies exploited by the parasite, to evade the defense mechanisms of the host, are also being considered as potent antileishmanial targets using immunomodulators. Immunomodulators are compounds capable of altering the response of the immune system and can behave either as immunostimulators or immunosuppressives [316]. Immunostimulators enhance the host response against infectious diseases or tumors by triggering pro-inflammatory cytokines or response [317]. Since *Leishmania* for successful survival subverts the host immune responses and defense machineries, use of such immunomodulators can prove a futuristic therapy against the disease. Fucoidan, the sulphated polysaccharide derived from *Fucus vesiculosus,* has long been used traditionally as an anti-thrombic and anti-malarial agent. Fucoidan was reported to be effective in controlling both antimony-susceptible and -resistant VL [318]. The antileishmanial effect was explained later by the study demonstrating that fucoidan treatment mediates PKC-dependent MAPK and NFκB activation, eventually leading to the production of NO and disease resolving cytokines [319]. Cystatin is another immunomodulator that inhibits cysteine proteases of *Leishmania* by which the parasite ensures a disease-conducive Th2 response in the host [320]. Cystatin treatment shifts the CD4+ cell differentiation from Th2 to Th1 and also upregulates NO production [321]. Later studies also indicated that cystatin treatment could effectively cure experimental VL by NFκB-mediated proinflammatory response in infected macrophages [322]. The list of immunomodulators studied against leishmaniasis include the triterperne derivative from the water-soluble extract from the roots of licorice −18-β-glyccyrrhetinic acid (GRA). GRA treatment activated NFκB in experimental VL animal models along with concomitant upregulation of NO production and Th1 cytokines [323]. Later studies by this group determined that GRA treatment also reversed the host phosphatase/kinase balance, which is altered by the parasite as a survival strategy [324]. Another bioactive component from the roots of licorice, glyccyrrhizic acid (GA), was also shown to exhibit antileishmanial properties. Treatment of *L. donovani*-infected macrophages with GA inhibited COX2-mediated prostaglandin E2 release in the host and increased expression of pro-inflammatory cytokines like IL-12 and TNF-α [325]. The authors suggested that GA treatment, in combination with SAG, was effective against SAG-resistant *L. donovani*. GA treatment inhibited expression and efflux activity of host ABC transporter multidrug resistance-associated protein-1 (MRP-1) and host P-glycoprotein (P-gp), which were responsible for efflux of antimony from SAG-resistant infected host cells [326]. Interestingly, a recent study reports an alternative antileishmanial mechanism of GA where GA treatment abrogated *L. donovani* growth by inhibiting *L. donovani* HMG-coA- reductase (HMGR), depleting membrane ergosterol levels [327]. A similar effect of GA has been reported very recently in CL. Treatment with GA and hydroalcoholic extract of *Glycyrrhiza glabra* inhibited the growth of promastigotes and amastigotes [328]. Another immunomodulator that has been very recently reported to possess antileishmanial potency is genipin. Genipin inhibits *L. donovani*-induced UCP2 levels and hence increases ROS generation in infected macrophages. Genipin treatment also successfully drove the cytokine balance to the disease resolving Th1 state. Genipin reduced splenic and liver parasite burden, despite showing no direct effect on both the promastigotes and axenic amastigotes [157]. Thus, the antileishmanial potency of genipin is by targeting the host-negative regulatory protein, UCP2, which is exploited by the parasite for ROS suppression. Like GA, combination of genipin and SAG showed synergism against leishmaniasis, suggesting that combination therapy involving such immunomodulators and other frontline antileishmanial drugs may be a promising futuristic antileishmanial approach.

## Concluding remarks

*Leishmania* as a parasite has evolved various mechanisms and strategies to overcome host defense machineries. The quest to find the effectors that makes the parasite capable of subverting host defense mechanisms led to the discovery of various virulence factors like LPG, EF1-α, and GP63. LPG and GP63 have been reported to play important roles in evading the primary defense artilleries of the host like ROS and RNS production, inhibiting NFκB activation, overriding different host signaling, and shifting the host immune response to the disease conducive Th2 state. The parasite as a survival strategy also exploits certain host-negative regulatory proteins like A20, UCP2, HO-1, Arginase, c-Myc, and SHP-1, but the mechanism or the factors responsible for these are not very well elucidated, and further works are required to answer these questions. With passing time and increased research in this area, novel virulence factors are coming into light like Hsp78, LACK, various leishmanial kinases, and deubiquitinases. Understanding of the leishmanial virulence factors and host immune subversion strategies has led to advanced diagnostics of the disease and has suggested futuristic drug therapies, which is the need of the hour in order to tackle the menace of leishmaniasis.

## Data Availability

All the relevant data (tables and figures) of this review are available.
